# Bibliometric Analysis: Insights Into the Podiatric Medicine Landscape of Diabetic Sensory Peripheral Neuropathy and Genomics

**DOI:** 10.1002/jfa2.70062

**Published:** 2025-07-24

**Authors:** Benjamin M. Jones, Reuben J. Pengelly, Alan M. Borthwick, Catherine J. Bowen

**Affiliations:** ^1^ School of Health Science University of Southampton Southampton UK; ^2^ Faculty of Medicine University of Southampton Southampton UK

**Keywords:** bibliometric, diabetes, genomics, peripheral neuropathy, podiatry

## Abstract

**Background and Aims:**

Research into diabetic foot complications is extensive; it remains challenging to identify critical literature. Evolving interprofessional boundaries, alongside advances in molecular medicine and pathophysiological understanding, necessitates mapping of the scientific literature (corpus). Impact of these advances on podiatric medicine remains unclear. This study explores topics, research performance, and evolution across the literature and disciplines to understand the corpus in its current state.

**Method:**

A retrospective‐observational bibliometric analysis examined Web of Science publications using PRISMA search strategy (August 2023) to understand interconnectedness, direction, and intersectionality of subject disciplines, growth areas, and output. Curated phrases and disease focussed classification anchored investigation to diabetic peripheral neuropathy. Qualitative and quantitative approaches analysed publication meta‐data (authors, citations, keywords) to map key concepts and scientific developments.

**Results:**

Analysis of 589 records (1991–2023) revealed observational studies as the dominant design. Prominent concepts included risk, polyneuropathy, and prevalence, with authors favouring accessible terms (peripheral neuropathy) across specialisms. Leading research hubs were in England, Demark, USA, Qatar, Germany, and Italy. Diabetic Medicine and Diabetes Care remained the highest‐cited journals, whilst the International Journal of Molecular Science, Cell Stem Cell, and Nature Reviews Neurology provided contemporary insights. Post‐2016, methodological rigour and objectivity increased.

**Discussion:**

Recurring topics included enhancing pre‐clinical screening, addressing earlier diagnosis, pain management stratification with medicines optimisation, and reproducibility challenges. Case‐controls increasingly replaced larger prospective, longitudinal study designs to improve diagnostic test accuracy and detection of diabetic neuropathy, particularly for neuropathic pain affecting small nerve fibres. Molecular approaches gained prominence signalling a shift from purely clinically derived approaches. The corpus responded to subjectivity and variable diagnostic criteria by prioritising objectivity. Emerging insights into channelopathies and mitochondrial dysfunction may augment current assessment/screening approaches to refine risk stratification and management strategies.

## 1 Introduction

Diabetic foot disease (DFD) is estimated to contribute 1.8% towards total global disease burden when using disability‐adjusted life‐years [1]. Peripheral neuropathy (PN), without ulceration or amputation, represents the largest proportion of DFD and receives limited attention [1]. International guidelines rely upon broad definitions for PN usually referring to nonspecific dysfunction, despite representing a core subpopulation that can receive preventative approaches if intervention is sufficiently early [2, 3]. This lack of detail to form or subtyping, for example, sensory over motor, constrains guideline value [4]; not all forms of peripheral neuropathy are equal. Guidelines do not reflect compelling evidence that characterisation of PN is necessary to improve diagnostics and prognostic approaches [5, 6, 7, 8, 9, 10]. The IWGDF 2023 risk stratification for ‘at‐risk of foot ulceration’ provides risk categorisation of 1 (low) when loss of protective sensation is identified reflecting progress towards differentiation of 0 (very low risk) [11, 12]. However, the spectrum between fully sensate and dysfunctional retains a ‘present or absent’ approach. Investigation into the scientific literature of diabetic foot complications (DFC) is needed to highlight topics, gaps and evolution to understand how approaches have integrated fields and expertise.

Previous bibliometric analysis in the field of DFD covered 1955–2023 with a variety of professional lenses [13, 14, 15, 16, 17, 18]. Diabetic limb salvage groups identified extensive volume and breadth for practitioners to traverse in updating the practice in 2012 analysis [18]. The sheer volume renders comprehensive reading impossible across the scientific corpus (the total body of scientific research dedicated to the subject matter); breadth of fields makes identification across disciplines difficult when appraising where to target first. Surgical management teams 1992–2010 identified intellectual structures on tissue integrity, restoration and management. Core themes, surgical‐management and diabetic journey‐related emerged from *n* = 1229 articles to *n* = 122 reviews; however, the research did not reflect the expanse of fields involved across the life course of patients living with diabetes (PLWD) [17]. Prevention and prediction have been slow to emerge in the corpus despite urgency to reduce DFC incidence; early action to prevent poor health outcomes (AR1 [area of research interest objective]) is a critical research priority for Department of Health and Social Care, UK [19]. Language and focus from surgical to multidisciplinary across these periods marked the transition from reactive to proactive approaches; migrating away from ‘diabetic foot’ ∼1955 to ‘diabetes‐related foot disease’ ∼2020, showing an evolving lexicon (language) seeking precision [13, 16].

Journals, ‘Diabetes Care’, ‘Diabetic Medicine’ and ‘Diabetologia’, have historically housed most DFC research [20]. Understanding where new research is emerging can highlight new structures within the corpus—USA, England, Germany and Italy have consistently been identified as high‐produces [17] alongside increasing output from China, Australia and the Netherlands [15]. The type of research produced globally can locate the contemporary nexuses of talent. Researchers and clinicians alike can draw on this expertise in a targeted fashion. To illustrate, an Australian‐only bibliometric analysis [14] (*n* = 322 articles; drawn only from Scopus) between 2012 and 2021 noted a five‐fold increase in their annual publication alone. Their evaluation of research types, using UKCRC Health Research Classification System, indicated progress with aetiology and treatment evaluation. Screening and diagnosis research remained low and prevention was virtually nonexistent [14]; the paucity may be a global phenomenon and not isolated to Australia. However, knowledge deficits inevitably necessitate clinicians and researchers to source from international research that may not transfer as readily to their local setting or population.

Finally, genetic research on diabetic foot ulcers (DFU) has seen an increased representation in lower limb‐centric journals (2007–2021) [21, 22]. DNA variation impacting DFU can include multiple body systems, for example, inflammatory‐centric, vascular and neurological, reflecting a wide range of research areas to digest [21, 23, 24]. Genetic risk scoring, for example, has been investigated in prevention to support assessment for type 2 diabetic patients through identification of genetic high‐risk susceptibility [25]. However, robust clinical translation has been mixed overall [26, 27, 28, 29], thus inviting discussion on how applications would impact DFC. Utilising one prominent DFC (that can yield preventative and prognostic potential), this study sought to understand where the research focus was occurring, the form and direction alongside evolution. Using a collection of research constituents (authors, institutions, countries) across the literature and disciplines, we employed a variety of bibliometric output metrics to assist in deriving topics and mapping knowledge domains to understand intellectual structures (prominent areas of focus) (Glossary of terms available in Supporting Information S1).

## 2 Materials and Method

Best practice guidelines were adhered to using PRIBA reporting [30]. To understand knowledge structures (conceptual, intellectual and social), use of performance and network analysis were mapped to meet the study's aims and objective. The underlying constituents were sourced from articles and reviews to analyse authors, keywords, citations, journals, institutions and countries (See Supporting Information S2 for source and metric).

### 2.1 Metric Selection

Performance metrics using total frequency, frequency per year, or averages across citations of papers, authors, keywords, keywords plus, or other relevant research constituents were chosen to identify highest performing elements (Further details Supporting Information S3). Summary of component mapping‐to‐objectives are found in Supporting Information S2: Table S1. Recommendations by Donthu et al. 2021 [31] were followed to ensure maximisation of breadth of techniques for analysis were employed.

### 2.2 Search Strategy

**FIGURE 1:**
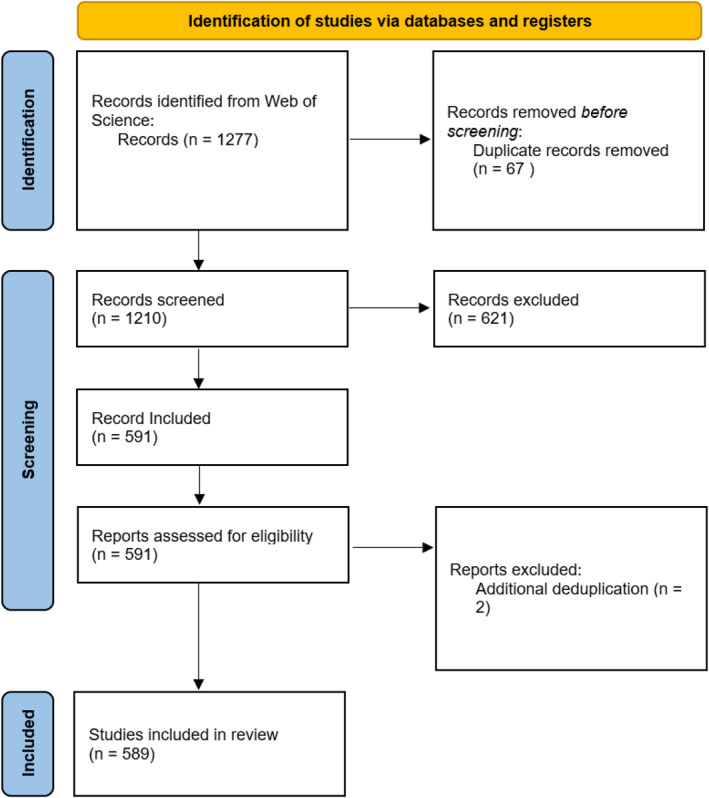
PRISMA workflow.

Exploration focused upon ‘no‐ or low‐risk’, as defined by NG19 (NICE Guidelines) [32], diabetic sensory peripheral neuropathy (DSPN) and corresponding neurological assessments with overlapping genomics. A curated selection, *n* = 15 papers (Supporting Information S4: Table S2), was used to define optimal keywords (see Table 1). The focus centred upon a key trait: Peripheral neuropathy within the diabetic population, across podiatry and genomics; search was restricted to include diabetic foot complications/ulcers to ensure anatomical preference in the lower limb. Common assessments or emerging modalities were used to help encapsulate risk/categorisation (See Supporting Information S4 Search Strategy Expanded for full breakdown). The approach sought a balance between stringency and inclusivity, achieving an initial dataset (*n* = 1277) (Figure 1). This approach overlapped with assessments for DFC, therefore formed the necessary boundaries of search, which included all forms of articles until final search on 31st August 2023.

**TABLE 1:** Included keywords/phrases selected with justifications: These shaped the bibliometric search for papers accounting for synonyms.

Keywords/phrases	Justification
Diabet* AND Peripheral Neuropath* OR Polyneuropath*	Podiatric centric implications Capture target population Early literature (established high‐ranking words) Capture target physiological measure
Sensory OR Diffuse OR Sensorimotor OR Distal OR symmetrical OR vibration	Capture target population Early literature (established high‐ranking words) Capture target physiological measure
Assessment OR Categorisation OR Categorization OR vibration OR quantitative testing	Broader literature (established high‐ranking words) Contemporary literature and preferred target Capture modalities use to evaluate peripheral neuropathy Reveal current topic in intellectual structures Delineate current state of prognostication
Genom* OR Gene* Or Polymorphism OR SNP OR Mutation* OR phenotyp* Peripheral Neuropath* (PN)	Genetic/genomic focus Developing literature (high‐ranking wording) Modern literature (accurate wording selection) Reveal current topic in intellectual structures
Risk OR Risk Profile OR Stratification OR Prognosis OR Prognostication OR personalised medicine	Modern literature (accurate wording selection) Delineate risk as an intellectual constituent Capture modalities use to evaluate peripheral neuropathy Reveal current ranking to risk categories Delineate current state of prognostication

*Note:* During analysis, phrases were critical when applying filters to understand and ensure capture of nuanced insight.

### 2.3 Study Selection

Single author [B.J.] screening utilised the Rayyan.AI software [33]. The screening author's previous clinical career was as a specialist podiatrist, thus able to draw upon clinical insights and application to verify relevancy of material to practice [34]. Inclusion criteria remained broad to capture assessments, basic science and genetic features (mutations or polymorphisms) alongside key phenotype of sensory neuropathy (regardless of form). Breadth of document type was desirable in discerning where research was located, for example, primary articles and reviews. Therefore, we included editorials, books, proceedings and abstracts, for example, conferences, short reports and reviews. Exclusion of case studies, case series and animal‐models occurred. Treatment‐centric publications within the inclusion criteria that conducted clinical neuropathic assessments using either quantitative sensory testing or perception thresholds were permissible where abstracts indicated relevancy. Exclusion further removed intervention‐only studies and drug induced peripheral neuropathy, for example, chemotherapy‐induced, as the cause of DSPN. Sources reporting off‐target but overlapping subjects, for example, Fabry and Gaucher Rare Diseases, or where nonhuman samples was reported, were also excluded.

The terms excluded or merged to improve analysis, for example, Fibre and Fibre, to provide accurate count during analysis are supplied within Supporting Information S11. The exclusion terms file removed core words/phrases where, as primary search terms as they would have been overrepresented (therefore trivial) (Supporting Information S6: Figures SF7–SF12 demonstrate the impact of filters upon results). This improved accessibility of emerging terms that had smaller representation, thus indicating new or hidden concepts as they entered top 10–30 (see Supporting Information S4: Tables ST4 and ST5). Filters were applied selectively during analysis only (appropriately delineated within relevant results section).

### 2.4 Data Preprocessing and Quality Control

We assessed for completeness of data and determined dataset to be robust (See Supporting Information S10; Figure S2).

#### 2.4.1 Database Selection

The Web of Science (WoS) repository was accessed: 31st August 2023. This database offered adequate biomedical coverage and provided optimal compatibility when intergrated with selected analytical software returing the most robust dataset. Cochrane, Len.org, Embase and Scopus databases were excluded as exported records lacked adequate quality to conduct analysis.

#### 2.4.2 Software

*Screening*: Rayyan.AI [33] software as a service (SaaS) Webform: Accessed on 25th August 2023. No version number available at time of use.

*Analysis*: Biblioshiny was the principle platform for analysis (Bibliometrix version 4.1.4, which integrated BIBLIOMETRIX [35] GEPHI [36] VOSviewer [37]).

### 2.5 Data Analysis

All findings were returned to the team [R.P., C.B., and A.B.] for discussion and interpretation. Performance metrics, for example, highest output, were reported to provide snapshot/summary over time and raise awareness of authors, subject matter expertise, and prominent papers to guide this analysis and discussion only. Descriptive analysis of performance (contribution), as noted in Donthu et al. (2021) [31], was selected to elucidate which authors held sway through metrics to identify core contributors that shaped the corpus and direction. Core sources (journals) were investigated to determine where publications resided to give sense to structure, further supported by employing ‘Bradford's Law’ to visualise their presence. Co‐occurrence and co‐citation provided insight to relationships between constituents and were explored analytically. The m‐index was selected for journals and authors to help identify contemporary value. This metric takes the Hirsch index (h‐index) and divides this by the number of years since the author’s first publication. A h‐index, briefly, measures how many authors publications have been cited as least as many time. The m‐index constrains time to help interpret the balance between publications, citations, and recency. It is intended to highlight contemporaneousness of authorship and improve comparrison against well‐established authors.

## 3 Results

### 3.1 Overview

The eligible dataset covered 1991–2023, with sample of *n* = 589 records containing *n* = 246 sources; articles were most prominent *n* = 424 (72%) followed by reviews *n* = 119 (20%). The breakdown of record characteristic can be found in Table 2. Exploring averages across document types, growth rate was 2.19% annually, document age ∼12 years and citations per document were 51.64 across *n* = 17,573 references. Total of *n* = 1414 ‘Keyword Plus’ terms were identified and *n* = 2550 authors. Of which, *n* = 38 (0.015%) were single‐authored documents. Co‐authorship per document was *n* = 6.14 and international co‐authorship reflected 25.47% of sample. Peak research article production occurred in 2021 with *n* = 44 (range 22–44) climbing gradually from 1993. The citation average per year peaked in 1993 with 12.5 (range 1–12.5) and from 1995 to 2021, the range of the citation average remained within 2–6 per year (*Data were not shown for peaks and citation average year*).

**TABLE 2:** Record characteristics and overview of records.

Description	Results
Main information about data	
Timespan	1991:2023
Sources (journals, books, etc.,)	246
Documents	589
Annual growth rate %	2.19
Document average age	12
Average citations per doc	51.64
References	17,573
Document contents	
Keywords plus (ID)	1414
Author's keywords (DE)	1022
Authors	
Authors	2550
Authors of single‐authored documents	38
Authors collaboration	
Single‐authored documents	45
Co‐authors per document	6.14
International co‐authorships %	25.47
Document types	
Article	424
Article; book chapter	7
Article; early access	4
Article; proceedings paper	19
Editorial material	2
Meeting abstract	8
Note	1
Proceedings paper	3
Review	119
Review; book chapter	1
Review; early access	1

A deeper exploration of highly cited and influential studies (*n* = 37) revealed that observational investigations were the most frequent approach. Subgroup characteristics consisted of: Cross‐sectional studies (13) [6, 38, 39, 40, 41, 42, 43, 44, 45, 46, 47, 48], cohort (9) [6, 38, 49, 50, 51, 52, 53, 54, 55], reviews (9) [8, 10, 56, 57, 58, 59, 60, 61, 62], epidemiology (6) [6, 38, 55, 56, 63, 64], case–control (3) [65, 66, 67], position statements (2) [68, 69], validation/other (2) [7, 9], guidelines (1) [70] and special reports (1) [71]. Classification of studies reflects the updated precision in study design and evidence‐based medicine [72, 73, 74, 75, 76, 77, 78, 79, 80]. A collation of common instruments, trials and guidance can be found in Table 3.

**TABLE 3:** Collation of instruments, major studies and consensus guides prevalent with the articles.

Instruments	Trials/major survey/national dataset
Michigan Neuropathy Screening Instrument (MNSI) Michigan Neuropathy Screening Instrument Examination part (MNSI‐E) Michigan Diabetic Neuropathy Score (MDNS) Neuropathy Diabetes Score [1980] [81] Neuropathic Disability Score Neuropathy Symptom Score Neuropathy Symptom Score [Modified] Neuropathy Symptom Profile [1986] [82] Total Neuropathy Score Neuropathy Total Neuropathy Score‐6 Toronto Clinical Scoring System Toronto Neuropathic Clinical Score (TNCS) Modified‐Toronto Neuropathic Clinical Score (TNCS) Utah Early Neuropathy Scale LANSS (Leeds Assessment of Neuropathic Symptoms and Signs) Overall Neuropathy Limitation Scale (ONLS) PainDETECT Douleur Neuropathique en 4 Questions (DN4) Neuropathic Pain Symptom Inventory (NPSI) Brief Pain Inventory (BPI)	Seattle Prospective Diabetic Foot Study Rochester Diabetic Neuropathy Study UK Prevalence Study 1993 EURODIAB IDDM Complications Study National Health and Nutrition Examination Survey Pain in Neuropathy Study (PiNS) German Diabetes Study Group Methods for early detection of diabetic peripheral neuropathy (MEDON) Danish National Patient Register UK Bioresource Rare Disease Project UK Primary Care CPRD Gold Database
Consensus guides (for diagnosis)	
San Antonio Consensus Criteria Mayo Clinic Classification Revised American Diabetes Association Recommendation	

### 3.2 Language, Subject Categories and Core Concepts

Keyword plus across 1991–2023 delineated the core concepts (See Table 4); higher frequency indicated strength within the corpus. Application of the synonym filter revealed risk (129) was greater in importance than polyneuropathy (104). Exclusion terms (alongside synonym filter) re‐ranked accordingly: Prevalence (99), complications (64), foot ulceration (51), diagnosis (48) and skin biopsy (38) (Supporting Information S4: Figure S3). Focus centred upon clinical presentation, umbrella terms and impact (See Supporting Information S4: Keyword Plus and Author Keywords). Subject categories (WoS) revealed clinical neurology and neuroscience contained the most relevant research followed by medicine general and internal, biochemistry and molecular biology, and anaesthesiology (Supporting Information S4: Figure S4).

**TABLE 4:** Top‐10 keyword plus and author keywords presented without filters applied to demonstrate “as is” state of the corpus.

Top‐10 keyword plus	Top‐10 author keywords
Peripheral neuropathy (184)	Diabetic neuropathy (84)
Polyneuropathy (103)	Neuropathy (79)
Prevalence (99)	Diabetes mellitus (58)
Risk‐factors (82)	Peripheral neuropathy (52)
Mellitus (73)	Diabetes (51)
Sensory neuropathy (67)	Quantitative sensory testing (49)
Complications (64)	Diabetic peripheral neuropathy (37)
Foot ulceration (51)	Skin biopsy (31)
Diagnosis (48)	Neuropathic pain (29)
Corneal confocal microscopy (44)	Small fibre neuropathy (29)

Author keywords presented a different focus (Table 4). These revealed modalities of quantitative sensory testing and skin biopsy were more prominent concepts pertaining to peripheral neurological assessment. Synonym filter consolidated concepts to rerank accordingly: Peripheral neuropathy (104), quantitative sensory testing (51), small fibre neuropathy (44) and painful neuropathy (38). Additional exclusion filter saw hidden concepts, diabetic foot (24), nerve conduction studies (16) and diagnosis (15) elevate in prominence. Authors centred on practical applications and approaches.

#### 3.2.1 Co‐Occurrence

Centrality metrics quantitatively demonstrate where connections convey information effectively (in volume, across fields and journals). High betweenness measures propagation of shared meaning. Prevalence, as a concept, is universally understood, indicating the corpus is aware of the health burden resulting from peripheral neuropathy alongside other complications, particularly DFU. Low centrality measures in primary health care and health care sciences and services revealed these intellectual structures underperformed comparative to high conveyers: Clinical neurology and neuroscience (See Table 5).

**TABLE 5:** Noteworthy centrality measures in keyword plus and subject categories.

Centrality measure	Centrality
Keywords plus (unfiltered top 5)	
Peripheral neuropathy	290.39
Polyneuropathy	75.20
Prevalence	55.04
Sensory neuropathy	50.04
Risk‐factors	39.04
Keywords plus (filtered top 5)	
Prevalence	323.52
Foot ulceration	62.25
Impaired glucose‐tolerance	50.18
Complications	49.82
Skin biopsy	47.90
Subject categories (top 3; filter not applicable)	
Clinical neurology	124.77
Neurosciences	122.22
Biochemistry and molecular biology	112.01
Subject categories (low 3; filter not applicable)	
Primary health care	3.64
Genetics and Heredity	0.0
Health care sciences and services	0.0

*Note:* When filters were applied to ‘keyword plus’, reranking occurred to reveal prominent concepts strongest in conveying information revealing ‘prevalence’ as the highest, alongside ‘foot ulceration’ and ‘complications’. In subject categories, top 3 were clinical neurology, neuroscience and biochemistry, and molecular biology revealing which subjects conveyed information best as a hub. This was important to compare against bottom 3, where we identified low communicators/conveyers of information; primary health care, genetics and heredity and health care sciences and services.

### 3.3 Authors and Papers

#### 3.3.1 Core and Emerging

**FIGURE 2:**
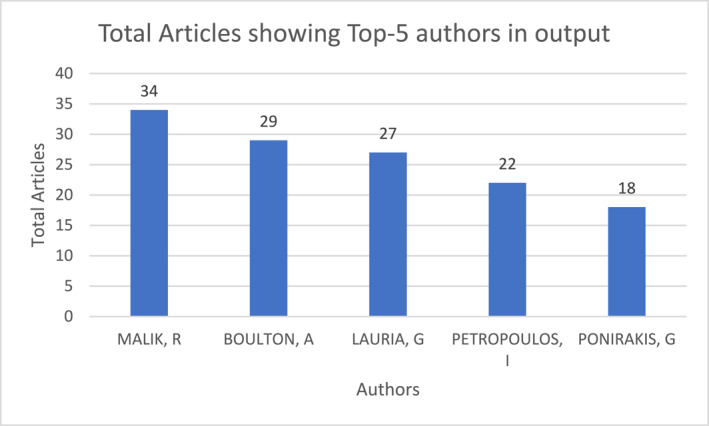
Total articles per author; top 5 present across 1991–2023.

**FIGURE 3:**
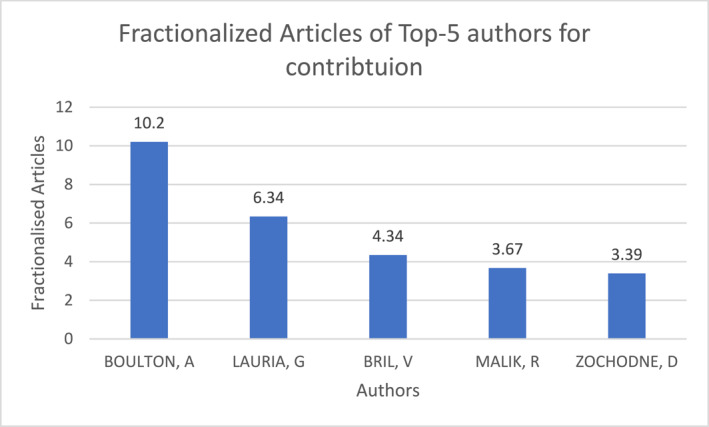
Fractionalised articles per author; top 5 present across 1991–2023.

Recurring authors Azmi, Boulton, Devigili, Dyck, Feldman, Lauria, Malik, Sumner, Tesfaye, Themistocleous, and Young were revealed through high output and preferred citations (within dataset; ‘local citations’) of prominent papers. First authors with established and recognised contribution became readily visible through output. Conversely, ‘global citations’ (counted citations within search WoS repository) revealed authors, Backonja, Dyck, Iqbal, Lauria, Rosenberger, Young, and Zaharia, to be influential beyond the local dataset. New entrants in ‘normalised local citations’ and m‐index global rate show distinct overlap with Croosu and Røikjer as recent influential contributors. Collectively, these authors shaped the corpus (See Supporting Information S4: Tables S7–S9 and S12). Total articles of the top‐5 authors introduced Petropoulos and Ponirakis as important contributors in output (Figure 2). Fractionalised articles of top‐5 authors introduced Bril and Zochodne (Figure 3). Boulton, Lauria and Malik remained commonly known figures.

**FIGURE 4:**
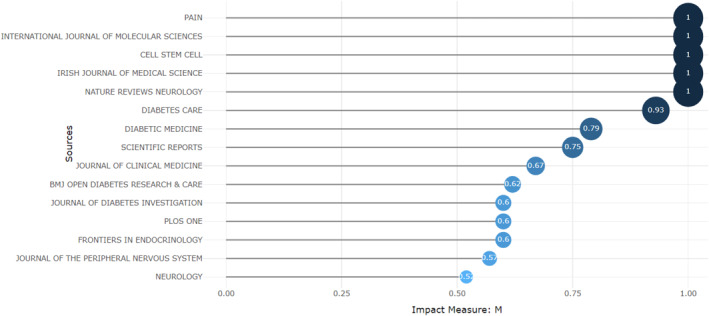
Sources impact by m‐index measure (within dataset); m‐index communicates relevance over time, thus an m‐index of pain (11) over an 11‐year period has an impact of 1, comparatively, m‐index of journal of peripheral nervous system (13) over a 23‐year period, has impact of 0.57. Overall range was 0.52–1.00: Recency this revealed the emergent knowledge structures. Critically, we see Cell: Stem cell and International Journal of Molecular Medicine outperform Diabetic Medicine and Diabetes Care. Neurology and pain high rate scores in m‐index impact measure, in their respective time frames, indicate increasing value. Recent publication (2022) by Ethiopian authors in the Irish Journal of Medicine accounted for the presence of our outlier in higher Irish Jorunal of Medical Science representation.

The citations using historiography (Figure 4) further highlighted Sumner, Devigili, Petropoulos and Ponirakis as influential authors (latter two from 2014 onwards). Author's most active periods (peak output or seminal work within dataset) have been tabulated (see Table 6).

**TABLE 6:** Summary of authors' production over time between 1991 and 2023.

Authors	Division (period)	Context and interpretation Citation per year refers to peak across authors
Malik and Boulton Young and Veves	1991–2023 1991–2013	Longest producing research across pertinent search terms. High citation paper in 1993 identified ‘earliest nexus’. Youngs' larger prospective study (1993–1994) built upon Dyck et al. platform [83]. Tesfaye et al. [10, 84, 85, 86] contributed significantly within this period. Boulton, Young and Veves occupied first half of period. Malik occupied second half.
Lauria and Sommer Polydefkis	2005–2022 2003–2018	Consistent output with high citations post‐2005. Lauria built upon McArthur et al. [51] seminal work in 1998 transform into core guidance (2008–2010) [44, 70] particularly with intraepidermal nerve fibre density. Sommer and Polydefkis overlapped in discrete periods sharing authorship.
Petropoulos, Ponirakis, Alam and Marshall Azmi and Ferdousi	2014–2023 2015–2021	Concentrated period of accelerating output, shared authorship, both 2015 and 2021 noted high citation articles. Malik output increased with 2018 and 2021 providing highly cited papers.

*Note:* Key authors in each period scored higher on a variety of performance metrics. Tranches provide insight into which authors help influence each period.

### 3.4 Journals

#### 3.4.1 Exploring Sources

**FIGURE 5:**
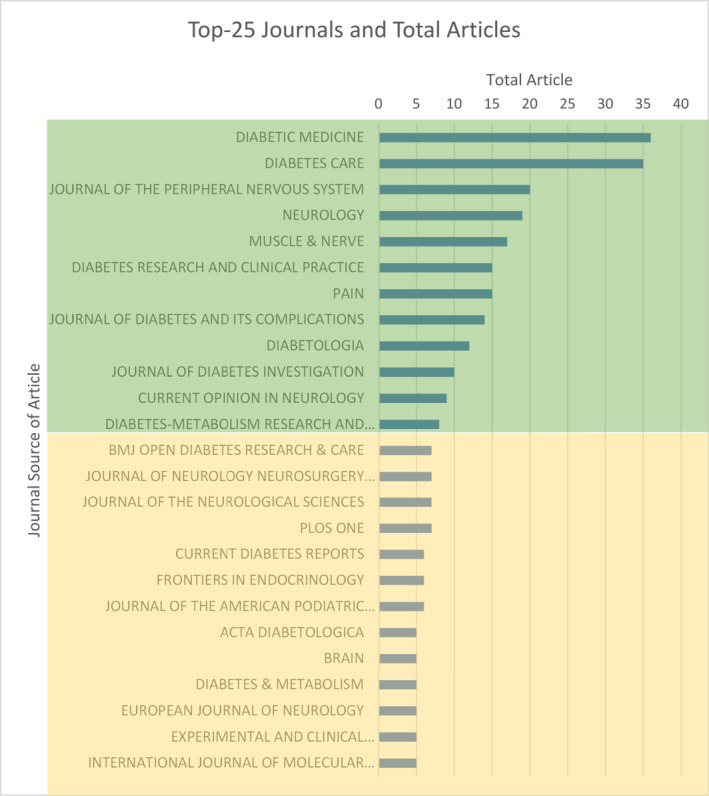
Across period 1991–2023 core sources (journals) represented main intellectual structure of research. Bradford’s law batches the cumulative frequency of highest performing journals. Green zone indicates core journals of key papers. Yellow zone represents influential journals (either increasing/decreasing relevant output), Diabetic Medicine (36) and Diabetes Care (35) represented the strongest pillars. Journal of peripheral neuropathy (20), neurology (19) and muscle and nerve (17) highlight promising intellectual structures.

The most established intellectual structures (sources) were Journals of Diabetic Medicine and Diabetes Care, suppling *n* = 71 key articles. The emergence of The journal of peripheral neuropathy (JPN), neurology, and muscle and nerve showed supporting structures where interest was expanding (See Figure 5; Supporting Information S4: Table S16).

The m‐index (See Figure 4) revealed journals of pain, molecular sciences, cell: stem cell, and nature reviews neurology achieved the highest rate of 1: High contemporaneousness. Diabetes Care (0.93), Diabetic Medicine (0.79), and JPN (0.57) rates were respectively lower. Focus on neuropathic pain and molecular components within these articles demonstrated increasing prominence closer towards 2023. Applications of genomics to prognostication, stratification, screening, and diagnosis within podiatric medicine were embryonic [87, 88].

Care and Medicine represented core sources persistent across the entire period. Figure 3 shows eminent fields (subject categories) providing highest cited literature associated to search terms.

#### 3.4.2 Co‐Citation

Network analysis of journals (Supporting Information S9; SF1) demonstrated two major themes (See Supporting Information S6: Figures S5 and S6):

1. Management of Diabetes (Clinic‐centric): Represented by journals Diabetes Care, Diabetic Medicine, Diabetologia, Diabetes Research Clinical Practice, New England Journal of Medicine, Diabetes and its Complication, Diabetes Metabolism Research and The Lancet.
2. Neuroscience (Scientific‐centric): Represented by neurology, muscle and nerve, pain, diabetes, brain, journal of neurology, neurosurgery and psychiatry, annual of neurology, peripheral nervous system and journal of neuroscience.

The concepts of brain, periphery, and pain became the prime focus of research inheriting both management and care legacy with journals neurology and diabetes care being the least insular sources.

### 3.5 Core Institutions

The ranked breakdown of institutions shows the concentration of productivity (Table 7) highlighting The University of Manchester being a major research hub alongside Harvard and Aalborg. Institutions in England, Denmark and USA presented the most influence in shaping the corpus. Italy, Qatar, Germany, and France all featured within the top 20 (Supporting Information S4: Table S22).

**TABLE 7:** Top 5 affiliations of authors.

Affiliation	Frequency of affiliation
University of Manchester	75
Harvard University	44
Aalborg University	41
Johns Hopkins University	37
Aarhus University	35

*Note:* N8 partnership had *n* = 55 but was removed as this was not a single institution adding confounding to results. Frequency of affiliation refers to the sum of authors times their institution. For The University of Manchester, across all papers from 1993 to 2023, shows 75 counts of authors associated to this institution. This helps identify research hubs for relevant research.

### 3.6 Core Output Across Periods

Influential papers were categorised into five tranches, each reflecting recurring topics, major trials and evolving research priorities:

1. 1991–1998; research focused on enhancing assessments and diagnosis. Large prospective trials in United Kingdom and United States of America (Rochester and Seattle) generated primary evidence, favouring cross‐sectional study designs. Nerve conduction and vibratory assessment featured more prominently, and research was driven by clinical need and expertise. Diabetes Care and Diabetologia were the leading journals.
2. 2001–2005; evolution towards evaluating and verifying these new assessments/approaches; smaller cohort studies began establishing preference towards quantitative sensory testing. The period responded to evidence heterogeneity, study designs, and quality. Reliance on consensus driven expertise alongside review studies for assessment, screening, and diagnostic criteria translated into practical guidance.
3. 2008–2015; saw invasive assessment skin biopsy emerge, with recommendation and guidance. Small‐fibre neuropathy became vogue. Increasingly, neurophysiology and neuroscience were contributing more evidence with clinical diabetes research giving way to clinical neurology. Peripheral nerve society and pain journal gained prominence.
4. 2016–2019; molecular medicine entered the scene and sensory phenotyping gained momentum. Painful neuropathy, prediabetes diagnosis, and glycaemic management for DFC prevention gained focus. Channelopathies and genetic association pertaining to small fibre neuropathy emerged and larger cohort studies were appearing from Germany.
5. 2020–2023; phenotype stratification was considered a viable approach to targeted pain management. Case–control studies became more prominent, but vastly smaller participant numbers compared with tranche 1 prospective studies. Point‐of‐care testing was more explicit and vibratory assessments re‐entered vogue. Translation medicine from Denmark and Netherlands introduced new approaches to remedy reproducibility challenges. Large public dataset, clinical health records and genetic repositories were increasing in visibility alongside scientific analysis at scale. A formal Delphi process enhanced expert consensus on assessment, classification, diagnostic criterion and management. Mitochondrial research alongside neuroscience was reflected by Molecular Sciences and Neural Transmission journals (See Supporting Information S5: Tables S24 and S25 for deeper breakdown).

### 3.7 Trends and Topic: Recurring, Emergent and Subtopic

- *Preventative approaches were underdeveloped/developing*
  ◦ Early detection desirable/attempted
  ◦ Remedy sought to rectify delayed detection/under‐diagnosis
- *Detecting preclinical signs remained challenging*
  ◦ Point‐of‐care/In situ methods lack consistency, variability in diagnostic criteria
  ◦ Absence of assessment focused randomised control trials (RCTs); investigative rigour missing
- *Clinical Management preference*
  ◦ Concentration of authors/institutions
  ◦ The need to diversify thought, population and setting
- *Nomenclature predominantly clinically‐centric*
  ◦ Neuroanatomical distribution remained the primary stratification approach
  ◦ Nuanced pathological detail missing/incomplete; better phenotyping needed
- *Reproducibility challenges persistent*
  ◦ Role reform in/across disciplines; research‐practice gap notable
  ◦ Standardisation lacking

Influential literature, highlighted by the historiography and high‐rate m‐index authors, was explored with greater scrutiny. Topics were discerned accordingly reflecting recurring frequency. Explicitly, literature reported the value in early diagnosis; contemporary articles and reviews expressed need for robust preventative strategies. Instrumentation, although comprehensive for general neurological assessment, favoured late‐stage manifestation in PLWD; early‐stage presentation remained difficult to capture. Standardised assessment application across research designs suffered from high heterogeneity and inconsistency. Randomised control trials comparing investigative approaches did not feature in top 20. Clinical manifestation shaped nomenclature and stratification, with authors relying upon these terms to categorise patients, in particular employing neuroanatomical distribution. Over the 30 year period, variations in reproducibility were due to differences in instrumentation and assessment thresholds. Evolving research in pain and painless neuropathy highlighted emerging use of molecular characterisation; however, preference using neuroanatomical distribution to classify DPN remained dominant. Subsequently, risk profiling and stratified management appeared as key priorities.

## 4 Discussion

### 4.1 Overview

This study successfully met our objectives by analysing a disease‐focused classification with preventative and prognostic potential. We mapped the research landscape by examining various constituents, authors, institutions and countries. Our findings chartered the existing landscape and illuminated emerging trends and gaps, providing a foundation for future research. Observational study design dominated the period; articles and reviews were most prevalent document types averaging an age ∼12 years since publication. Prospective, longitudinal and cohort trial studies [6, 39, 41, 50] exerted a strong influence within the corpus, potentially obscuring newer technologies and study designs [89, 90]. Given the extent of uncertainty between measures, reliance upon dated research and design preference does raises ethical concerns [91]. Liu et al. (2019) [92] noted similar trend, with 14 of 16 studies in their meta‐analysis using cross‐sectional designs, therefore deriving causal relationships is challenging using observational studies [93, 94], potentially accounting for slow progress across fields and period. As such, value and influence should be appreciated cautiously and may account for alternative research design selection.

From 2020, case–control design gained traction in subgroup discrimination (e.g., nonpainful vs. painful neuropathy) [48, 67]. Reviews, position and consensus statements [57, 68] relied heavily on established researcher hubs to consolidate emergent trends [8, 10, 56, 57, 58, 59, 60, 61, 62] and shape diagnostic criterion [10, 56] reflecting conceptualisation, progression and mobilisation of key author knowledge and influence. However, unstructured reviews weakened their value [8, 10, 56, 60, 62], prompting more rigorous Delphi method by Ziegler et al. [57]. Scottish Intercollegiate Guidelines Network (SIGN) 116 were updated last in 2017 [95], NICE (UK) Diabetic Foot Guidelines [NG19] in 2019 [96], American and Australian in 2021 [97, 98] and IWGDF in 2023 [11, 12]. The American, Australian and IWGDF reflect partial elements in language and tools; however, disagreement remains in low/no‐risk screening approaches [99, 100]. Appraisal update of America guidelines identified future research pathway into painful DPN and recognised importance of DPN prevention [100]. UK clinical guidelines should integrate modern research findings [32, 101], particularly as new classification systems emerge, integrating pathophysiological understanding, to refine sensory profiling, risk categorisation and reduce vagueness [53, 102] (especially within low/no‐foot risk groups). To address experimental evidence deficit, international, multicentre and randomised control trials (RTCs), leveraging cutting edge‐techniques (e.g., molecular subtyping [58]) are necessary to reduce uncertainty but appear lacking in this field.

Most instruments relied upon symptom scoring (Table 3). The absence of validated clinical instruments focused on lower limb and sensory aspects was notable. Although systematic approaches (e.g., quantitative sensory testing [QTS]) offer structure for replication [103, 104], they remain time‐consuming and may not capture preclinical symptoms effectively. Validated instrumentation could standardise data collection in health records, supporting pattern recognition to aid earlier risk stratification. Despite their value in assisting assessment [10, 57, 68], instruments are not yet embedded in guideline recommendations [11, 12]. The persistent gap in reliable point‐of‐care screening indicates an incomplete capacity for early risk detection.

Keyword analysis revealed recurring concepts of risk, prevalence and complications (Table 4), emphasising the difficultly in achieving meaningful diagnostic and screening solutions. Western‐centric population cohort were overrepresented [39, 49, 54, 105, 106], limiting generalisability of risk factors research into DPN. Corneal confocal microscopy, despite stringent publication screening to exclude, was well‐represented, reinforcing its diagnostics potential in small fibre neuropathy [107, 108, 109, 110]. Surprisingly, lower limb‐centric concepts were underrepresented, suggesting broad terminology persists where precise, nuanced classification may enhance risk stratification. Without standardised foot and ankle diagnostic tool (with equivocal performance), risk classification will remain nonspecific.

Authors preference for general terms (e.g., diabetic neuropathy) over granular subdivision (e.g., small fibre neuropathy) (Table 4), suggest prioritisation of accessibility over specificity. Centrality measures demonstrated shared understanding across fields (Table 5), although helpful for interdisciplinary collaboration, greater precision in terminology could support clearer specificity of focus therefore enhance staging and progression DPN models [24, 111, 112, 113, 114], thus reduce uncertainty.

**FIGURE 6:**
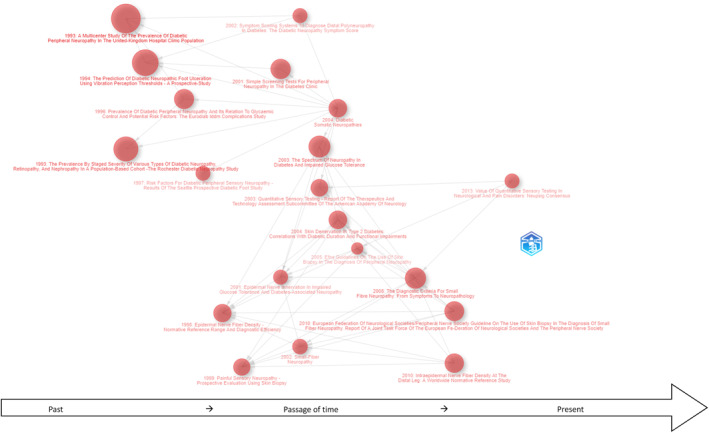
Historiography showing document titles and directed graph of referencing. Each node shows document and citation influence with arrow highlighting which document referenced one another. Larger nodes were cited most across local and global datasets. Evolution of topics demonstrates journey through vibration perception thresholds (1994), risk‐factors (1997), epidermal nerve fibre density (1998), screening (2001), quantitative sensory testing (2003), skin biopsy (2005), small nerve fibre (2008), peripheral nerve biopsy (2010) and quantitative sensory testing (2013) [in pain].

Analysis identified formal research hubs and influential authors [6, 8, 9, 10, 39, 40, 41, 43, 44, 50, 53, 54, 56, 59, 62, 65, 70, 115], revealing stable and emerging contributors offering new research direction [47, 48, 67]. Prominent contributors reflected cross‐field links and shared focus [107, 108, 109, 110, 116, 117, 118, 119, 120, 121, 122, 123, 124, 125, 126, 127, 128, 129], revealing interface between concepts and collaborations. Emergent authors highlighted new research direction/contributions [47, 48, 67]. Highly cited research (Figure 6) reflected corpus preference towards scientific approaches nearer 2023; co‐citation network analysis visualising journals relationships (Figure S8) demonstrated increased neuroscience and peripheral nerve focus as core sources. Diabetes management and neuroscience were the overarching groupings for journals, the latter favouring reproducible methodologies. The USA, England and Germany have consistently been the leading contributors, whereas Denmark emerged as a noteworthy contributor (Table 7) (Canada, Italy and Qatar also resided within top 10). European studies increasingly prioritised early DSPN diagnosis [48, 60], with international consensus on definitions developing from ∼2010 [10]. Contemporary publications resided within pain, molecular sciences, cell: stem cell and nature reviews neurology focusing upon pain management and pathological mechanism [46, 59, 60], addressing subjectivity, heterogeneity (presentation) or variable diagnostic criterion [2, 68, 69].

Methodological advancements from 1998 onwards included intraepidermal nerve density sampling [44, 51] (Figure 6) and corneal confocal microscopy, in particular for small fibre neuropathy [65], marking a shift from invasive diagnostics. Consequently, then shaping detection and pain management approaches, for example, medicines optimisation [57, 58, 59, 60, 100]. By 2022, revised QST and molecular characterisation aimed to improve classification and screening accuracy [48, 67]. The period post‐2016, reflected adoption of novel technologies to refine polyneuropathy classification. Emphasis upon neural transmission, dysfunction and form of diabetes impacted neuromodulation and neuroregeneration [130]. Recent research suggests neurological‐specific biobanking and next‐generation sequencing (NGS) (e.g., single cell sequencing), could standardise diagnostic approaches, remedy QST limitation and enable better reproducibiligty [131]. Despite its dominance, monofilament testing remains controversial likely due to reliability concerns [132, 133], thus emphasising the need for consistent quantification to guide screening and cost‐effectiveness assessments.

### 4.2 Podiatric Relevance

Management of DFU necessitates use of multidisciplinary teams (MDTs) [134, 135, 136, 137], with podiatrists playing a central role in screening, risk classification, monitoring, management and prevention [134, 138, 139, 140, 141]. However, the lack of standardised sensory phenotyping complicates accurate risk assessment [142], forcing specialists to wait for late‐stage confirmatory manifestation.

Emerging risk prediction tools offer potential solutions [143, 144, 145]. Haque et al. [143] nomogram‐based severity predictor (97.95% accuracy) leveraged lower‐limbs assessments, drawn from Michigan Neuropathy Screening Instrument (MNSI), but no podiatric‐specific tool attained prominence within research corpus. Existing instruments, primarily from neurology and neuroscience, may need to be repurposed; results herein reveal an abundance of research instruments to select from (Table 3). Effective screening must move beyond neuroanatomical distribution ensuring tools.

- mitigate perception biases [47]
- maintain high accuracy [48, 67], and
- enable non/minimally invasively point‐of‐care assessment

National guidelines lack instrumentation and prediction tool recommendations (however, nomograms have not yet been validated), suggesting a disconnect between pertinent research and practice [11, 12]. Incorporating such measurements may provide liable data to address bias towards monofilament testing. This also suggests that vibration testing remains underutilised, despite significant sensitivity in prediabetic population [57, 60].

#### 4.2.1 Implication to Screening, Monitoring, Diagnostics and Assessment Approaches

The absence of optimal screening and establish diagnostic scrutiny continues to contribute to DPN underdiagnosis [57, 146]. Novel approaches, such as functional MRI for pain discrimination, remain impractical for community settings [47, 147]. However, genetic screening for channelopathies could identify high‐risk individuals (anticipated to experience severe symptoms) to receive early intervention [46, 54, 148], whereas whole‐genome sequencing (WGS) may further refine aetiological classification across peripheral neuroapathies [45, 54]. Lower‐limb specialists could integrate genetic data interpretation into clinical monitoring, enhancing early detection and risk stratification.

Vibratory assessments featured prominently as a concept (Supporting Information S6: Figure S13) and within influential publications. Of which, ∼16% (*n* = 6) [38, 39, 40, 42, 50, 52], were explicitly reported with consistent methodology. Comparatively, ∼13% (*n* = 5) [38, 39, 40, 52, 56] employed QST, which included both pressure (monofilament) and autonomic assessment; despite widespread use, monofilament lacked clear conceptual distinction, likely attributable to sensitivity concerns [149], inconsistent testing site [150] and scrutiny over thresholds [49, 132]. Vibratory assessment value vacillated between tantalising ‘screening tool’ [50], ‘limited value’ [151] and ‘important for early‐stage disease’ [57, 60]. Development of normative reference for age likely improved categorisation utility [40, 57, 152]; future thresholds require country or ethnicity‐specificity to improve accuracy [153]. The Ipswich touch test (IpTT) [154, 155, 156, 157] was noticeably absence, reinforcing the corpus preference for objective over subjective measures despite recommended screening tool in IWGDF [11, 12]. Emerging approaches, such as vibration threshold tracking (VTT) in Denmark [67], show promise as they navigate issues of neural accommodations that adapt during extended testing [103, 104]. However, before clinical implementation can occur, consensus on standardisation is required.

### 4.3 Comparison to Contemporary Work

This bibliometric analysis aligns with Zha BS et al. 2019 [22] and Deng et al. 2022 [158], confirming key contributing countries and topics. Recent work by Tang et al. [86] similarly identified dominant topics of risk factors, epidemiology, diagnosis and complications with genetic research gaining importance [24, 58, 86, 111]. The PROTECT Study Survey 2 [159] identified podiatrists as key players in neurological assessments, research into structured deep phenotyping could standardise these approaches [160, 161]. In turn, helping to unmask insights currently inaccessible in clinical records to amplify ‘Omic investigations [160, 161, 162, 163] where ongoing challenges persist [164, 165].

### 4.4 Limitations

The exploratory design precluded in‐depth paper analysis, limiting explanatory depth. Citations, a crude metric, cannot reflect quality or impact [166, 167]. All performance metrics should be viewed cautiously. Single screening author limited generalisability; however, this was partially offset through findings discussion (consensus between authors) to taper inherent bias. Institutions were disambiguated by the software; therefore, it is possible that some were either over or under counted. Using a single database may have caused us to have incomplete publication capture. Future bibliometric designs should consider which research constituents offer the highest informational yield, pertinent to these fields, to inform revised bibliometric protocol design [166]. Topics were manually extracted via single author [BJ] with additional research team input upon finding; full systematic thematic analysis was not employed at this juncture.

### 4.5 Conclusion

The explored topics, contextualised through synopsis, revealed high desirability for early identification and prevention of DPN. Reproducibility issues have prompted multiple fields (diabetes care and neurology) towards objectifying assessments; focus is upon improving screening and clinical risk prediction in PLWD to minimise DFU. Pre‐2016 period was predominantly clinically‐led research; post‐2016, migration occured towards scientific‐led research designs. This likely reflected new approaches to diagnostics and risk categorisation. Sources revealed subject matter involving molecular medicine, biochemistry and stem cells—particularly for neuropathic pain management for small fibre neuropathy, are of growing interest clinically for medicines optimisation. Further investigation into molecular‐augmented assessments/screening or clinical tools/instrumentation linked with preventative approaches could address areas where paucity of evidence was noted.

## Author Contributions

**Benjamin M. Jones:** conceptualization (equal), date curation (equal), formal analysis (lead), investigation (lead), methodology (equal), project administration (lead), resources (lead), software (lead), supervision (equal), validation (equal), visualisation (lead), writing – original draft (lead), and writing – review and editing (equal). **Reuben J. Pengelly:** conceptualization (supporting), data curation (equal), formal analysis (supporting), methodology (equal), resources (supporting), software (supporting), supervision (equal), validation (equal), visualisation (supporting), writing – review and editing (equal). **Alan M. Borthwick:** supervision (equal) and writing – review and editing (equal). **Catherine J. Bowen:** conceptualization (equal), methodology (equal), supervision (equal), and writing – review and editing (equal).

## Conflicts of Interest

Benjamin M. Jones and Reuben J. Pengelly declare no conflicts of interest. Catherine J. Bowen and Alan M. Borthwick are emeritus editors of the Journal of Foot and Ankle Research. C.J.B. is supported by a National Institute of Health Research (NIHR) Senior Investigator Grant (#203706). The views expressed in this publication are those of the authors and not necessarily those of the NIHR.

## Supporting information

Supporting Information S1

Supporting Information S2

Supporting Information S3

Supporting Information S4

Supporting Information S5

Supporting Information S6

Supporting Information S7

Supporting Information S8

Figure S1

Figure S2

Table S23

## Data Availability

The authors have nothing to report.
