# Supplementary material for: Bibliometric Analysis: Insights Into the Podiatric Medicine Landscape of Diabetic Sensory Peripheral Neuropathy and Genomics
Source: J Foot Ankle Res. 2025 Jul 24;18(3):e70062. doi: 10.1002/jfa2.70062 (PMC12289441; doi:10.1002/jfa2.70062)
Supplement: Supplementary file 1 — Supporting Information S1 [file JFA2-18-e70062-s002.docx]

# Supplementary File 1 Glossary

**Conceptual Structure [CS]:** What science discusses; main themes/topics and trends e.g., co-words.

**Intellectual Structure [IS]:** How work of authors influences given scientific community e.g. Journal.

**Social Structures [SS]:** How authors, institutions, countries interact e.g., collaboration.

**Research Constituents [RS]:** Meta-data/data that make part of the paper e.g., Authors, Keywords, Citations, Institutions.

**Corpus:** Body of research housing all relevant fields, structures, and constituents.

**Knowledge Domain [KD]:** Where structures intersect and share meaning i.e., neighbourhood housing intellectual and social structures.

**Performance Analysis [PA]:** Supposition is output and reuse of materials metrics e.g., citations, assume value in crude quantitative terms.

**Science Mapping [SM]:** Intellectual interactions and structural connections of research constituents i.e., shape of research field.

**Network Analysis [NA]:** Assist determination importance of publication For example: well-connected work fundamentally links across fields and categories or seminar work that propagates new direction or increased productivity.

**Node:** Refers to constituent being used as an anchor to help determine a relationship.

**Domain Analysis:** Refers to overall collection data, sources (journals), authors (affiliations and countries), documents (contents and bibliographies), and clustering of the aforementioned

**Dataset:** Refers n=589 documents investigated in this study (unless otherwise specified)

**Local:** Dataset compared within only e.g., citations across included n=589 articles only

**Global:** Dataset compared database e.g., citations recorded from Web of Science database

**Research Constituents [RC]:** Refers to underlying components (authors, institutions, countries, journals)

**M-Index:** Extension of H-Index where time is constrained to help improve relevancy of performance metrics. Metric strives to counteract long-standing or overrepresented publications that might skew performance where more recent publications have not had the benefit of a long time horizon.

**DFC:** Diabetic Foot Complication

**DFU:** Diabetic Foot Ulcer

**DPN:** Diabetic Peripheral Neuropathy

**NGS:** Next Generation Sequencing

**WGS:** Whole Genome Sequencing

**IENFD:** Intraepidermal Nerve Fibre Density

**DTA:** Diagnostic Test Accuracy

**PLWD:** Patients Living with Diabetes

**ROC:** Receiver Operating Characteristics
