# Supplementary material for: Bibliometric Analysis: Insights Into the Podiatric Medicine Landscape of Diabetic Sensory Peripheral Neuropathy and Genomics
Source: J Foot Ankle Res. 2025 Jul 24;18(3):e70062. doi: 10.1002/jfa2.70062 (PMC12289441; doi:10.1002/jfa2.70062)
Supplement: Supplementary file 2 — Supporting Information S2 [file JFA2-18-e70062-s007.docx]

# Supplementary File 2 Aims, Objective, and Metric Alignment

## Aim

- To explore topics, performance, and evolution of research constituents across the literature, disciplines, and scientific knowledge to assist mapping knowledge domains to understand intellectual structures.

## Objectives

1. Explore and contextualise:
   1. Reoccurring topics present across the literature derived from scientific language.
   2. Evolution and prevalence of terms or phrases across the period throughout relevant fields.
   3. Bibliographic coupling to understand references, subject categories, field connections, and evolution across the period.
2. Explore publication metrics across domains to account for representation of current research direction and influential publications.
3. Explore where science output is most prevalent and consistent to determine where nexus of activity across research fields reside.
4. Summarise bibliometric data presented to provide conceptual and intellectual structure of domains and discover emerging trends.

Supplementary Table 1 Objective, Metric and Source, and Knowledge Synthesis contribution: Each objective has components mapped to the analysis and the contribution to overall results

| **Objective** | **Metrics and source (unit of analysis) under investigation** | **Insight Contribution to Knowledge Synthesis across Domains** |
| --- | --- | --- |
| 1.1 | Sources: keywords plus (words), co-words (keywords), titles, abstracts, year of publication (documents)  Metric: Frequencies; Normalisations: Square Root | Conceptual Structures  Performance Analysis  -Co-occurrence |
| 1.2 | Sources: keywords plus, co-words (keywords), author keywords (documents)  Metric: Frequencies, Year |  |
| 1.3 | Sources: keywords plus, co-words (keywords), Most relevant (including citation totals and averages) and co-citation, journals, references, year of publication  Metric: Frequencies, centrality, Bradford’s Law,  Normalisation: Association  Network Clustering: Walktrap | Intellectual Structures  Scientific Mapping  Network Analysis  -Co-citation  Historiography |
| 2 | Sources: countries, countries of authors, journals, references,  Metrics: total citations (normalised), reference citations, production over time, co-authorship, M-Index (journals) | Performance Analysis  Social Structures  Scientific Mapping |
| 3 | Sources: countries, countries of authors, institutions, journals  Metrics: total publications, production over time, proportion of co-authorship | Performance Analysis  Scientific Mapping  Social Structures  -Collaboration Network |
| 4 | Convergence and blending of insights from metrics and approximate structure design | |
