# Supplementary material for: Bibliometric Analysis: Insights Into the Podiatric Medicine Landscape of Diabetic Sensory Peripheral Neuropathy and Genomics
Source: J Foot Ankle Res. 2025 Jul 24;18(3):e70062. doi: 10.1002/jfa2.70062 (PMC12289441; doi:10.1002/jfa2.70062)
Supplement: Supplementary file 3 — Supporting Information S3 [file JFA2-18-e70062-s004.docx]

# Supplementary File 3 Methodology Expansion

## Data Pre-processing and Quality Control

Reliability of meta-data were reviewed before analysis using Biblioshiny completeness of data. Manual curation of authors names occurred where duplication would impact ranking. Core metrics had low missing data with none exceeding 10% loss overall except ‘keywords’ with 26.83%. Keyword plus offset this, having greater validity as a measure of informational value, by having only 5.77% loss. Data was determined to be robust overall and suitable for further analysis [See Supplementary Figure 2 for breakdown].

## Metric Selection

Performance metrics provided a crude weighting to importance. Reflected predominantly using, frequencies e.g. total citations/keywords, and averages (or normalised transformations e.g. M-index). Keywords Plus was preferred as an algorithmic derivative index^156^, briefly: This metric reduces, from documents and references, core terms that best represent the *unit of analysis* resulting in more objective selection (comparative to author keywords that have been curated with intent)^157^. Mapping was assisted by demonstrating interconnectivity (relationships) and positionality of constituents^158^. Therefore, a blend of metrics included frequencies, centrality measures (betweenness, closeness, PageRank), co-presentation e.g., co-citation, and year of publication. Network analysis provided a statistical approach to cluster underlying components after normalisation e.g., association followed by Walktrap (*Detecting communities in a language co-occurrence network using ‘short distant random walks’. These merge groups until communities form; supposition is short walks remain in a similar community^159^*). Summary of component mapping-to-objectives is found in Supplementary File Aims, Objective, and Metric Alignment. Recommendations by Donthu et al, 2021^31^ were followed to ensure maximisation of breadth of techniques for analysis to interrogate the literature to create a comprehensive picture.

## DORA Principles

Responsible use of performance metrics aligned to DORA principles^160^: Metrics e.g., highest output of authors or institutions, provided a transient snapshot of output to identify a nexus of expertise to guide investigation. Normalised approaches provided more comparable quantification, but were contextualised against multiple factors. Intent of metrics was to assist identifying influential work and collaborations to appreciate corpus evolution; insights are constrained to this dataset and are not absolute but relative markers in time.

**Position Statement:**

In accordance with DORA principles, metrics selected that may construe performance are explored in the context of corpus shape and density of output. Indexes within bibliometrics lack generalisability and are subject to bias, influence, or misinterpretation. Focus has remained upon Transparency, Equality, Appropriateness, Reproducible, and Continuous Reassessment. PRIBA Framework used to guide reporting^30^.
