# Supplementary material for: Bibliometric Analysis: Insights Into the Podiatric Medicine Landscape of Diabetic Sensory Peripheral Neuropathy and Genomics
Source: J Foot Ankle Res. 2025 Jul 24;18(3):e70062. doi: 10.1002/jfa2.70062 (PMC12289441; doi:10.1002/jfa2.70062)
Supplement: Supplementary file 4 — Supporting Information S4 [file JFA2-18-e70062-s005.docx]

# Supplementary File 6 Search Strategy Expanded

## Search Strategy

Supplementary Table 2 Curated sources n=15 derived literature keywords/phrases: A prior study selection to capture representation of fields, nomenclature and minimise irrelevant data.

| **Curated literature to source keywords** |
| --- |
| 1. Sensory phenotype and risk factors for painful diabetic neuropathy: a cross-sectional observational study^161^ 2. Sensory-Motor Mechanisms Increasing Falls Risk in Diabetic Peripheral Neuropathy^162^ 3. Usefulness of the vibration perception thresholds measurement as a diagnostic method for diabetic peripheral neuropathy: Results from the Rio de Janeiro type 2 diabetes cohort study^163^ 4. Worldwide Research Trends on Diabetic Foot Ulcers (2004–2020): Suggestions for Researchers^152^ 5. Patterns of Nerve Conduction Abnormalities in Patients with Type 2 Diabetes Mellitus According to the Clinical Phenotype Determined by the Current Perception Threshold 6. Schwann Cells as Crucial Players in Diabetic Neuropathy 7. Low and High Frequency Vibration Perception Thresholds Can Improve the Diagnosis of Diabetic Neuropathy 8. Proteasome Modulator 9 Gene rs14259 Polymorphism in Patients with Diabetic Polyneuropathy 9. Neuropathy in prediabetes: does the clock start ticking early? 10. Diabetic Neuropathies Endpoints in Clinical Research Studies 11. Redefining distal symmetrical polyneuropathy features in type 1 diabetes: a systematic review Practice Parameter: The Evaluation of Distal Symmetric Polyneuropathy: The Role of Laboratory and Genetic Testing (An Evidence-Based Review) 12. Neurophysiologic findings in diabetic neuropathy: nerve conduction studies, electromyography, quantitative sensory testing 13. Differences and Similarities in Neuropathy in Type 1 and 2 Diabetes: A Systematic Review 14. Genetic and Epigenomic Modifiers of Diabetic Neuropathy 15. The natural history of diabetic peripheral neuropathy determined by a 12 year prospective study using vibration perception thresholds |

Supplementary Table 3 Excluded Phrases and Keyword Selection with relevant justification: Approach ensured bibliometric search for papers removed off-target results. During analysis these were critical when filters were applied to segments understand nuanced insight. For example, the word clouds demonstrated improved clarity and assurance of themes not being diluted, akin to specificity.

| **Excluded Keywords / Phrase** | **Justification** |
| --- | --- |
| Rat* OR Mice OR Animal Model  Fabry or Gaucher Disease (Rare)  Streptozotocin-induced OR Streptozotocin  Parkinson* OR Amyloidosis | If the study design had animal and human component e.g., human cell line comparison, these were retained for screening and determine on an individual basis otherwise animal modelling was to remove from principal research focus.  Rare diseases would confound research and introduce authors and citation form peripheral field not pertinent to this investigation. |

Various combinations were employed in different sequences to capture overlap, using advanced search terms combined with Boolean modifiers. A layering process was required to prevent aggressive filtering of relevant items e.g., ‘podiat*’ reduced most results to under 20 obstructing meaningful bibliometric analysis. Results were compiled within a ‘marked list’ on the Clarivate [Web of Science] platform with additional filters/constraints applied to enhance result relevance e.g., removal of studies where Alcohol* was considered a primary risk factor

Advanced Search Terms Combined with Boolean modifiers (Web of Science):

**((ALL=(vibration perception threshold* AND diabet*)) AND ALL=(diabetic peripheral neuropath*)) NOT ALL=(mouse OR mice OR rodent OR rate OR murine OR mouse model) [1983-2023]***

**(ALL=(diabetic peripheral neuropath*)) AND ALL=(genetics OR gene OR genes OR genomics) [1989-2023]***

**(((((ALL=(diabet* AND peripheral neuropath* AND sensory)) AND ALL=(risk OR risk profile* OR categorisation OR categorization OR assessment* OR stratification OR prognosis OR prognostication)) AND ALL=(polyneuropath* OR distral OR diffuse Or symmetrical Or small nerve fibre)) NOT ALL=(Rat* OR Mice OR Animal OR Animal Model OR Corneal OR Primate*)) AND ALL=(evaluate OR evaluation )) AND ALL=(diabetic foot complications OR diabetic foot ulcers) [1999-2023]***

**(((((ALL=(Diabet* AND Peripheral Neuropath* OR Polyneuropath*)) AND ALL=( Sensory OR Diffuse OR Sensorimotor OR Distal OR symmetrical OR vibration )) AND ALL=(Genom* OR Gene* Or Polymorphism OR SNP OR Mutation* OR phenotyp* Peripheral Neuropath* (PN))) AND ALL=(Assessment OR Categorisation OR Categorization OR vibration OR quantitative testing )) AND ALL=(Risk OR Risk Profile OR Stratification OR Prognosis OR Prognostication OR personalised medicine OR personalized medicine)) AND ALL=(podiat*) [2002-2022]***

***[] highlight what the range of years was for the records returned**

Prior to deduplication results were n=1277 (without limits to year); deduplicated 1210 post screening and application of eligibility criteria resulted

## Screening

Removal of any studies centric to:

- Corneal OR Confocal Microscopy (unless they had a vibratory or quantitative pressure testing component).
- Alcohol* OR chemotherapy* OR Charcot (neuro)Arthropathy OR Charcot-Marie-Tooth (as the exposure/outcome were not the focus).
- Treatments (which included most RCTs as these compared interventions); exceptions made to those that had a testing component of peripheral neuropathy or;
  - Attempted classification using key physiological measure (relevant to this research) or;
  - Involved human Stems cells iPSC or hPSC where development and maintenance of small nerve fibre, Swann, or relevant component to sensory perception.
- Certain rare disease e.g., Fabry were excluded to prevent confounding, however other conditions e.g., diabetic patients with HIV were only excluded if the study design did not align to principle investigation i.e., peripheral neuropathy, genomics, and podiatry.

## Keyword Plus and Authors Keywords


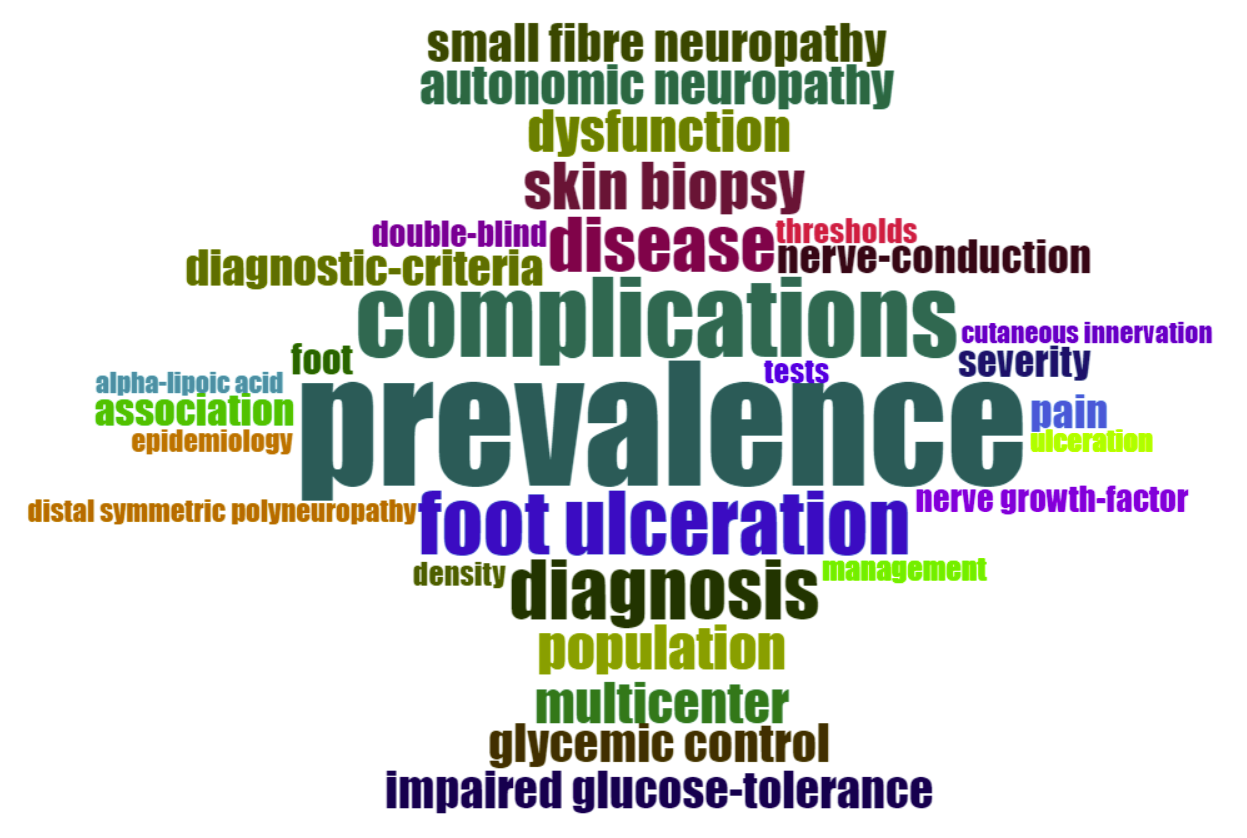


Supplementary Figure 3 Top 30 Keyword Plus word occurrence by frequency with filters applied: Prevalence, Complications, and Foot Ulceration


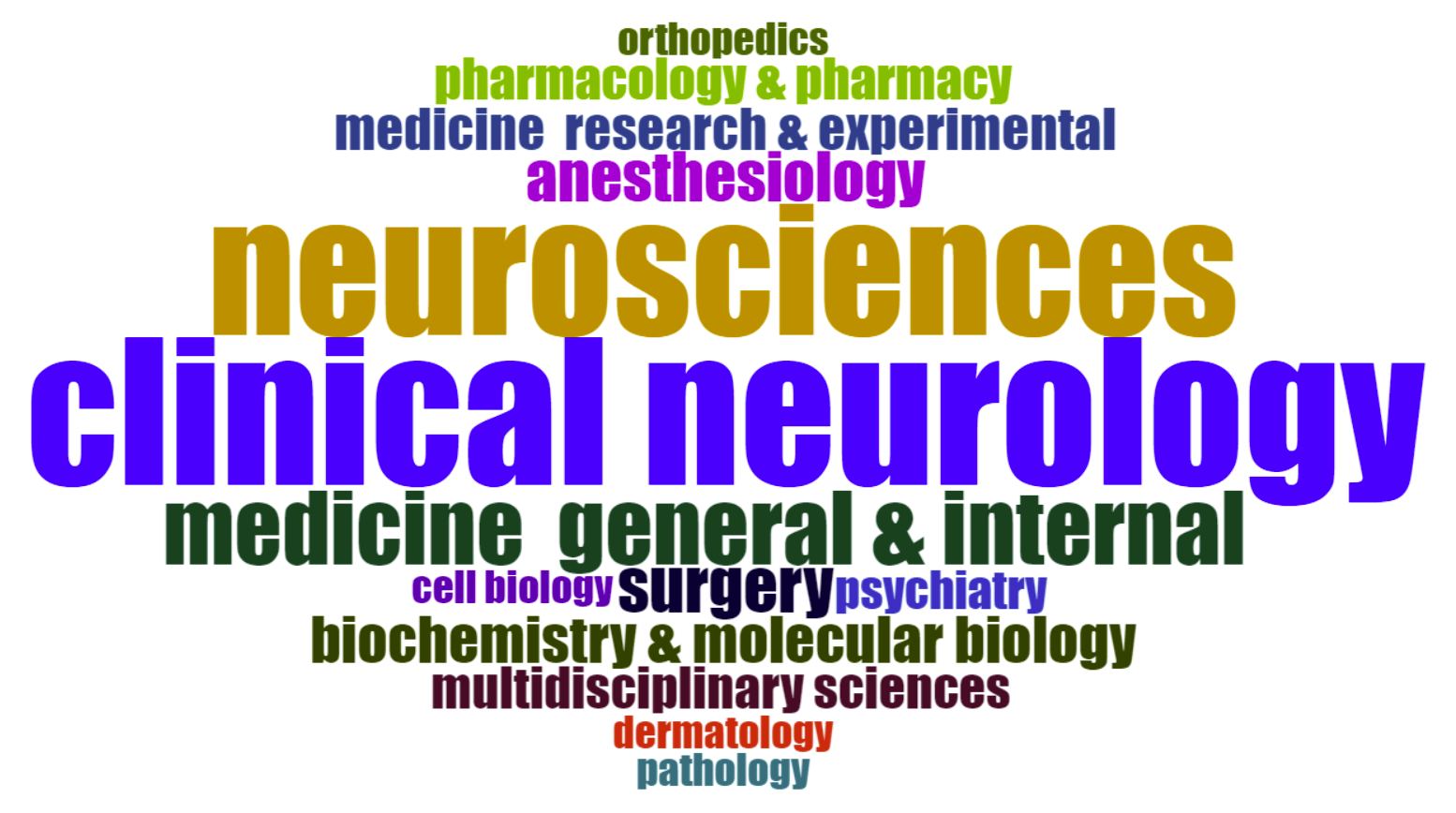


Supplementary Figure 4 Top 15 Web of Science (Subject Categories) word occurrence by square root: Clinical Neurology, Neurosciences, Medicine general & internal have highest keywork frequency across documents. Biochemistry & molecular emerged within top 10.

Supplementary Table 16 and Supplementary Table 17 show top 25 keyword plus and authors keywords respectively. Web of Science categories were minimally impacted by filtering and not included. Filtering layers demonstrated lexicon as overt terms were removed and duplication in meaning consolidated. Pre-filter provided popular terms and frequencies offer crude performance. Peripheral or diabetic neuropathy, prevalence and risk are persistent in high frequencies indicator of core themes.

Supplementary Table 4 Keyword plus top 25: Risk-factors, Risk, Peripheral neuropathy, and prevalence represent core constituents of corpus. Here, focus and language is centred upon biological components. Application of filters shows how prevalence, complication, and foot ulceration are the current reoccurring themes but sit on the peripheral of the knowledge. Sensory neuropathy is a core theme and close frequency to complication suggest they are linked within theme.

| Keyword Plus | | | | | | |
| --- | --- | --- | --- | --- | --- | --- |
| No Filter | | | **Synonym Filter Only** | | **Both Filters** | |
| Words | **Occurrences** | | **Words** | **Occurrences** | **Words** | **Occurrences** |
| peripheral neuropathy | | 184 | peripheral neuropathy | 191 | prevalence | 99 |
| polyneuropathy | | 103 | risk | 129 | complications | 64 |
| prevalence | | 99 | polyneuropathy | 104 | foot ulceration | 51 |
| risk-factors | | 82 | prevalence | 99 | diagnosis | 48 |
| mellitus | | 73 | mellitus | 73 | disease | 44 |
| sensory neuropathy | | 67 | sensory neuropathy | 67 | skin biopsy | 38 |
| complications | | 64 | complications | 64 | population | 35 |
| foot ulceration | | 51 | diabetic neuropathy | 57 | dysfunction | 34 |
| diagnosis | | 48 | foot ulceration | 51 | multicenter | 33 |
| corneal confocal microscopy | | 44 | diagnosis | 48 | autonomic neuropathy | 32 |
| disease | | 44 | corneal confocal microscopy | 44 | glycemic control | 31 |
| neuropathy | | 42 | disease | 44 | small fibre neuropathy | 31 |
| risk | | 42 | neuropathy | 42 | impaired glucose-tolerance | 30 |
| diabetic-neuropathy | | 40 | skin biopsy | 38 | diagnostic-criteria | 29 |
| skin biopsy | | 38 | population | 35 | nerve-conduction | 27 |
| population | | 35 | dysfunction | 34 | pain | 27 |
| dysfunction | | 34 | multicenter | 33 | association | 26 |
| multicenter | | 33 | autonomic neuropathy | 32 | severity | 25 |
| autonomic neuropathy | | 32 | glycemic control | 31 | foot | 24 |
| glycemic control | | 31 | small fibre neuropathy | 31 | double-blind | 21 |
| impaired glucose-tolerance | | 30 | impaired glucose-tolerance | 30 | nerve growth-factor | 21 |
| diagnostic-criteria | | 29 | diagnostic-criteria | 29 | tests | 20 |
| nerve-conduction | | 27 | nerve-conduction | 27 | thresholds | 20 |
| pain | | 27 | pain | 27 | density | 19 |
| association | | 26 | association | 26 | management | 19 |
|  | |  |  |  |  |  |

Supplementary Table 5 Authors keywords top 25: Diabetic neuropathy, Diabetes mellitus, Peripheral neuropathy, and Neuropathy represent core constituents of corpus from the authors perspective. Application of filters show how diabetes and diabetic are key identifiers, authors focus upon the condition. Quantitative sensory testing emerged as top 5. As the strangle filters are added focus has shifted into constituents associate to anatomy or assessment.

| Authors Keywords | | | | | |  |
| --- | --- | --- | --- | --- | --- | --- |
| No Filter | | **Synonym Filter Only** | | **Both Filters** | |  |
| Words | **Occurrences** | **Words** | **Occurrences** | **Words** | **Occurrences** |  |
| diabetic neuropathy | 84 | diabetes mellitus | 111 | small fibre neuropathy | 44 |  |
| neuropathy | 79 | diabetic neuropathy | 104 | painful neuropathy | 39 |  |
| diabetes mellitus | 58 | neuropathy | 79 | type 2 diabetes | 32 |  |
| peripheral neuropathy | 52 | peripheral neuropathy | 52 | skin biopsy | 31 |  |
| diabetes | 51 | quantitative sensory testing | 51 | diabetic foot | 24 |  |
| quantitative sensory testing | 49 | small fibre neuropathy | 44 | nerve conduction studies | 16 |  |
| diabetic peripheral neuropathy | 37 | painful neuropathy | 39 | diagnosis | 15 |  |
| skin biopsy | 31 | diabetic peripheral neuropathy | 38 | pain | 13 |  |
| neuropathic pain | 29 | type 2 diabetes | 32 | autonomic neuropathy | 11 |  |
| small fiber neuropathy | 29 | skin biopsy | 31 | electrophysiology | 11 |  |
| diabetic foot | 24 | diabetic foot | 24 | epidemiology | 11 |  |
| diabetic polyneuropathy | 24 | diabetic polyneuropathy | 24 | type 1 diabetes | 11 |  |
| diabetic neuropathies | 20 | vibration perception threshold | 22 | screening | 10 |  |
| vibration perception threshold | 20 | risk | 21 | prevalence | 9 |  |
| risk factors | 18 | nerve conduction studies | 16 | complications | 8 |  |
| type 2 diabetes | 17 | diagnosis | 15 | nerve conduction | 8 |  |
| nerve conduction studies | 16 | polyneuropathy | 14 | diabetes complications | 7 |  |
| diagnosis | 15 | pain | 13 | inflammation | 7 |  |
| type 2 diabetes mellitus | 15 | autonomic neuropathy | 11 | monofilament | 7 |  |
| polyneuropathy | 14 | electrophysiology | 11 | amputation | 6 |  |
| pain | 13 | epidemiology | 11 | biomarker | 6 |  |
| autonomic neuropathy | 11 | type 1 diabetes | 11 | foot ulcer | 6 |  |
| electrophysiology | 11 | screening | 10 | intraepidermal nerve fiber density | 6 |  |
| epidemiology | 11 | prevalence | 9 | mitochondria | 6 |  |

### Language Evolution

*Keyword Plus* and *Author Keywords* highlight terms in use. Figure 5 shows the travel direction, demonstrating when terms were favoured, and showing the evolving focus. Areas under investigation within the early period 1993-2002 showed *prevalence, prospective,* and *clinical* had the highest centricity (power to communicate meaning); 1997-2002 saw a shift to *diabetic peripheral sensory neuropathy, screening, diagnosis,* and *distal polyneuropathy*. By 2001-2008 *nerve innervation, small fiber neuropathy, quantitative sensory testing, the spectrum of neuropathies, skin biopsy,* and *diagnostic criteria* were newly prominent. Finally, from 2008-2013 *intraepidermal nerve fiber density* and *quantitative sensory testing represented* contemporary research focus. Lexicon (language) within *titles* denotes broader and accessible terms to assist audience interest. The drift toward assessments, instrumentation, and measurement highlights where universal terms e.g., peripheral neuropathy, struggled in definitive taxonomy. An underlying relationship between improving understanding of human biology, risk-factors, and neuroanatomical distribution were reflected in the corpus. Deeper characterisation was common them throughout contemporary research e.g., increasing discrimination between Sensory Neuropathy and Neuropathic pain.

Supplementary Table 6 Trend topics of Top 50 Keyword Pus (1997-2023) and Top 30 Author Keywords (1991-2022): Unequal and overlapping tranches reflect broader shifts in nomenclature. The evolution of nerve terminology became increasingly more precise in Keyword plus. Across Keywords Plus and Author Keywords 3 snapshots of term usage are presented: Nerve conduction, Amputation, and Complication(s) used throughout 1991-2009 transformed to Vibration Threshold Perception, Foot Ulceration, Distal symmetric polyneuropathy (DSP), Small Fiber Neuropathy (SFN), and Diagnosis within 2002-2018. From 2016-2023 DSP and SFN were resident terms; Expression, Validation, and Protocol had replaced older phrases. In Author keywords, terminology retained a general and humanistic stance with focus upon contemporary clinical issues e.g., Painful Neuropathy. In 2002-2018 we see only a fleeting presence of Inflammation and Pre-diabetes. By 2016-2023 SFN and Biomarkers became more established. Complications remained the favoured term across the entire period. Keyword plus reflects the increasing scientific terminology we see more prevalent in basic science e.g., inflammatory-related pathophysiology.

| **Period ‘Snapshots’** | **Keyword plus Top 50 from 1997-2023** | **Author keywords Top 30 from 1991-2022** |
| --- | --- | --- |
| 1991-2009 | Semmes-Weinstein monofilament, NIDDM*, Lower-extremity amputation, Amputation, Peripheral nerve function, Complications, Variability | Sensory threshold, Foot ulceration, Electrophysiology, Nerve conduction velocity |
| 2002-2018 | Vibration perception thresholds, Tuning fork, Foot ulceration, Complications, Nerve conduction, Prevalence, Diagnosis, Distal symmetric polyneuropathy, Small-fibre neuropathy Variability | Electrophysiology, Nerve conduction studies, Peripheral nervous system diseases, Prevalence, Pre-diabetes, Inflammation, Complication, Painful neuropathy |
| 2016-2023 | Distal symmetric polyneuropathy, Small fibre neuropathy, Inflammation, Validation, Expression, Variability, Protocol | Complications, Small fibre neuropathy, Painful neuropathy, Type 1 diabetes, Biomarkers |

Note 2 *NIDDM = Non-insulin Dependent Diabetes Mellitus: Tranches were guided by author lens and other metrics: Cumulative Output of Journals [Sources] (1991-1999, 2000-2011, 2012-2023), Per Year Output of Journals [Sources] (1991-2000, 2001-2006, 2007-2023), Author Production over time (1991-2000, 2001-2013, 2014-2023), and Reference Publication Year Spectroscopy (1991-2003, 2004-2013, 2014-2023) which provided similar cut-offs to shape time slices. As Keywords Plus and Author Keywords offered more insight to lexicon of the corpus, trend topics presented the logical tranches. Overlap was unavoidable to highlight transitional nature of the language.

## Analysis

### Document

Supplementary Table 7 Top-5 Most Cited Documents using normalized citations. Local and global dataset divisions. Lead author, year, journal, frequency, and article title.

| **Most Cited Document** | | | | | | | | | |
| --- | --- | --- | --- | --- | --- | --- | --- | --- | --- |
| **Local (Normalized Total Citations)** | | | | | **Global (Normalized Total Citations)** | | | | |
| **Lead Author** | **Year** | **Journal** | **N** | **Article Title** | **Lead Author** | **Year** | **Journal** | **N** | **Article Title** |
| Røikjer | 2023 | Pain | 14.67 | Perception threshold tracking: validating a novel method for assessing function of large and small sensory nerve fibers in diabetic peripheral neuropathy with and without pain | Zaharia | 2019 | Lancet (Diabetes & Endocrinology) | 8.86 | Risk of diabetes-associated diseases in subgroups of patients with recent-onset diabetes: a 5-year follow-up study |
| Themistocleous | 2016 | Pain | 9.71 | The Pain in Neuropathy Study (PiNS)  a cross-sectional observational study determining the somatosensory phenotype of painful and painless diabetic neuropathy | Iqbal | 2018 | Clinical Therapeutics | 8.29 | Diabetic Peripheral Neuropathy: Epidemiology, Diagnosis, and Pharmacotherapy |
| Devigili | 2008 | Brain | 8.50 | The diagnostic criteria for small fibre neuropathy: from symptoms to neuropathology | Backonja | 2013 | Pain | 7.28 | Value of quantitative sensory testing in neurological and pain disorders: NeuPSIG consensus |
| Croosu | 2023 | Diabetes Care | 7.33 | Alterations in Functional Connectivity of Thalamus and Primary Somatosensory Cortex in Painful and Painless Diabetic Peripheral Neuropathy | Rosenberger | 2020 | Neural Transmission | 7.25 | Challenges of neuropathic pain: focus on diabetic neuropathy |
| Azmi | 2015 | Diabetes Care | 6.38 | Corneal Confocal Microscopy Identifies Small-Fiber Neuropathy in Subjects With Impaired Glucose Tolerance Who Develop Type 2 Diabetes | Lauria |  | European Journal of Neurology | 7.20 | European Federation of Neurological Societies/Peripheral Nerve Society Guideline on the use of skin biopsy in the diagnosis of small fiber neuropathy. Report of a joint task force of the European Fe-deration of Neurological Societies and the Peripheral Nerve Society |

Supplementary Table 8 Top-5 Most Cited Documents using total citations. Local and global dataset divisions. Lead author, year, journal, frequency, and article title.

| **Most Cited Document** | | | | | | | | | |
| --- | --- | --- | --- | --- | --- | --- | --- | --- | --- |
| **Local (Total Citations)** | | | | | **Global (Total Citations)** | | | | |
| **Lead Author** | **Year** | **Journal** | **N** | **Article Title** | **Lead Author** | **Year** | **Journal** | **N** | **Article Title** |
| Young | 1993 | Diabetologia | 110 | A multicentre study of the prevalence of diabetic peripheral neuropathy in the United Kingdom hospital clinic population | Young | 1993 | Diabetologia | 1083 | A multicentre study of the prevalence of diabetic peripheral neuropathy in the United Kingdom hospital clinic population |
| Young | 1994 | Diabetes Care | 83 | The Prediction of Diabetic Neuropathic Foot Ulceration Using Vibration Perception Thresholds: A prospective study | Dyck | 1993 | Neurology | 1047 | The prevalence by staged severity of various types of diabetic neuropathy, retinopathy, and nephropathy in a population‐based cohort  The Rochester Diabetic Neuropathy Study |
| Dyck | 1993 | Neurology | 70 | The prevalence by staged severity of various types of diabetic neuropathy, retinopathy, and nephropathy in a population‐based cohort  The Rochester Diabetic Neuropathy Study | Lauria | 2010 | European Journal of Neurology | 584 | European Federation of Neurological Societies/Peripheral Nerve Society Guideline on the use of skin biopsy in the diagnosis of small fiber neuropathy. Report of a joint task force of the European Fe-deration of Neurological Societies and the Peripheral Nerve Society |
| Sumner | 2003 | Neurology | 51 | The spectrum of neuropathy in diabetes and impaired glucose tolerance | Boulton | 2004 | Diabetes Care | 574 | Diabetic Somatic Neuropathies |
| Devigil | 2008 | Brain | 47 | The diagnostic criteria for small fibre neuropathy: from symptoms to neuropathology | Sumner | 2003 | Neurology | 518 | The spectrum of neuropathy in diabetes and impaired glucose tolerance |

Supplementary Table 9. Top-5 Most Cited Documents using citations per year for global dataset only. Lead author, year, journal, frequency, and article title.

| **Most Cited Document** | | | | |
| --- | --- | --- | --- | --- |
| **Global (Total Citations per year)** | | | | |
| **Lead Author** | **Year** | **Journal** | **N** | **Article Title** |
| Lauria | 2010 | European Journal of Neurology | 36.50 | European Federation of Neurological Societies/Peripheral Nerve Society Guideline on the use of skin biopsy in the diagnosis of small fiber neuropathy. Report of a joint task force of the European Fe-deration of Neurological Societies and the Peripheral Nerve Society |
| Zaharia | 2019 | Lancet (Diabetes & Endocrinology) | 35.43 | Risk of diabetes-associated diseases in subgroups of patients with recent-onset diabetes: a 5-year follow-up study |
| Young | 1993 | Diabetologia | 32.82 | A multicentre study of the prevalence of diabetic peripheral neuropathy in the United Kingdom hospital clinic population |
| Dyck | 1993 | Neurology | 31.73 | The prevalence by staged severity of various types of diabetic neuropathy, retinopathy, and nephropathy in a population‐based cohort  The Rochester Diabetic Neuropathy Study |
| Devigili | 2008 | Brain | 28.33 | The diagnostic criteria for small fibre neuropathy: from symptoms to neuropathology |

Supplementary Table 10 Top-25 documents ranked by normalised total citations (globally).

| **Paper** | **Normalized TC** |
| --- | --- |
| ZAHARIA OP, 2019, LANCET DIABETES ENDO | 8.86 |
| IQBAL Z, 2018, CLIN THER | 8.29 |
| BACKONJA M, 2013, PAIN | 7.28 |
| ROSENBERGER DC, 2020, J NEURAL TRANSM | 7.25 |
| LAURIA G, 2010, EUR J NEUROL | 7.20 |
| DEVIGILI G, 2008, BRAIN | 6.75 |
| THEMISTOCLEOUS AC, 2016, PAIN | 5.82 |
| MARTIN CL, 2014, DIABETES CARE | 5.74 |
| YOUNG MJ, 1994, DIABETES CARE | 5.33 |
| LACOMIS D, 2002, MUSCLE NERVE | 5.05 |
| TESFAYE S, 1996, DIABETOLOGIA | 4.98 |
| HERMAN WH, 2012, DIABETIC MED | 4.82 |
| VERDUGO RJ, 2022, CLIN NEUROPHYSIOL | 4.78 |
| AMIN N, 2016, WORLD J DIABETES | 4.58 |
| BARON R, 2023, NAT REV NEUROL | 4.58 |
| BOULTON AJM, 2004, DIABETES CARE | 4.42 |
| BOYKO EJ, 1999, DIABETES CARE | 4.39 |
| TERKELSEN AJ, 2017, LANCET NEUROL | 4.22 |
| JENSEN TS, 2021, BRAIN | 4.13 |
| PERKINS BA, 2001, DIABETES CARE | 4.11 |
| LAURIA G, 2005, EUR J NEUROL | 4.08 |
| ALBERS JW, 2014, CURR NEUROL NEUROSCI | 3.98 |
| ADLER AI, 1997, DIABETES CARE | 3.94 |
| LAURIA G, 2010, J PERIPHER NERV SYST | 3.91 |
| SUMNER CJ, 2003, NEUROLOGY | 3.76 |

Supplementary Table 11 Top-25 documents ranked by normalised total citation (locally).

| **Document** | **Year** | **Normalized Local Citations** |
| --- | --- | --- |
| ROIKJER J, 2023, PAIN | 2023 | 14.67 |
| THEMISTOCLEOUS AC, 2016, PAIN | 2016 | 9.71 |
| DEVIGILI G, 2008, BRAIN | 2008 | 8.50 |
| CROOSU SS, 2023, DIABETES CARE | 2023 | 7.33 |
| AZMI S, 2015, DIABETES CARE | 2015 | 6.38 |
| BACKONJA M, 2013, PAIN | 2013 | 6.31 |
| TESFAYE S, 1996, DIABETOLOGIA | 1996 | 6.00 |
| YOUNG MJ, 1994, DIABETES CARE | 1994 | 5.98 |
| SOPACUA M, 2019, J PERIPHER NERV SYST | 2019 | 5.88 |
| BURGESS J, 2021, DIAGNOSTICS | 2021 | 5.68 |
| LAURIA G, 2010, EUR J NEUROL | 2010 | 5.51 |
| LAURIA G, 2010, J PERIPHER NERV SYST | 2010 | 5.24 |
| PONIRAKIS G, 2020, DIABETES-METAB RES | 2020 | 5.16 |
| PERIQUET MI, 1999, NEUROLOGY | 1999 | 5.15 |
| MARTIN CL, 2014, DIABETES CARE | 2014 | 5.01 |
| RAPUTOVA J, 2017, PAIN | 2017 | 4.85 |
| MCARTHUR JC, 1998, ARCH NEUROL-CHICAGO | 1998 | 4.49 |
| LAURIA G, 2005, EUR J NEUROL | 2005 | 4.46 |
| PETROPOULOS IN, 2018, DIABETES METAB J | 2018 | 4.43 |
| BLESNEAC I, 2018, PAIN | 2018 | 4.43 |
| PONIRAKIS G, 2019, J DIABETES INVEST | 2019 | 4.41 |
| HERMAN WH, 2012, DIABETIC MED | 2012 | 4.39 |
| PERKINS BA, 2001, DIABETES CARE | 2001 | 4.37 |
| MALIK RA, 2011, DIABETES-METAB RES | 2011 | 4.27 |
| JENSEN TS, 2021, BRAIN | 2021 | 4.26 |

#### References

Supplementary Table 12 Top-5 Most Cited references using citations per year for global dataset only. Lead author, year, journal, frequency, and article title.

| **Most Cited References** | | | | |
| --- | --- | --- | --- | --- |
| **Local (Total Citations per year)** | | | | |
| **Lead Author** | **Year** | **Journal** | **N** | **Article Title** |
| Tesfaye | 2010 | Diabetes Care | 114 | Diabetic neuropathies: update on definitions, diagnostic criteria, estimation of severity, and treatments |
| Young | 1993 | Diabetologia | 110 | A multicentre study of the prevalence of diabetic peripheral neuropathy in the United Kingdom hospital clinic population |
| Young | 1994 | Diabetes Care | 83 | The prediction of diabetic neuropathic foot ulceration using vibration perception thresholds: a prospective study |
| Feldman | 1994 | Diabetes Care | 76 | A practical two-step quantitative clinical and electrophysiological assessment for the diagnosis and staging of diabetic neuropathy |
| Dyck | 1993 | Neurology | 70 | The prevalence by staged severity of various types of diabetic neuropathy, retinopathy, and nephropathy in a population-based cohort: the Rochester Diabetic Neuropathy Study |

Supplementary Table 13 Top-25 cited references ranked by citations within local dataset.

| **Cited References** | **Citations** |
| --- | --- |
| TESFAYE S, 2010, DIABETES CARE, V33, P2285, DOI 10.2337/DC10-1303 | 114 |
| YOUNG MJ, 1993, DIABETOLOGIA, V36, P150, DOI 10.1007/BF00400697 | 110 |
| YOUNG MJ, 1994, DIABETES CARE, V17, P557, DOI 10.2337/DIACARE.17.6.557 | 83 |
| FELDMAN EL, 1994, DIABETES CARE, V17, P1281, DOI 10.2337/DIACARE.17.11.1281 | 76 |
| DYCK PJ, 1993, NEUROLOGY, V43, P817, DOI 10.1212/WNL.43.4.817 | 70 |
| POP-BUSUI R, 2017, DIABETES CARE, V40, P136, DOI 10.2337/DC16-2042 | 56 |
| SHAMOON H, 1993, NEW ENGL J MED, V329, P977, DOI 10.1056/NEJM199309303291401 | 55 |
| BOULTON AJM, 2005, DIABETES CARE, V28, P956, DOI 10.2337/DIACARE.28.4.956 | 54 |
| DYCK PJ, 1988, MUSCLE NERVE, V11, P21, DOI 10.1002/MUS.880110106 | 53 |
| SUMNER CJ, 2003, NEUROLOGY, V60, P108, DOI 10.1212/WNL.60.1.108 | 51 |
| QUATTRINI C, 2007, DIABETES, V56, P2148, DOI 10.2337/DB07-0285 | 50 |
| TESFAYE S, 2005, NEW ENGL J MED, V352, P341, DOI 10.1056/NEJMOA032782 | 50 |
| DEVIGILI G, 2008, BRAIN, V131, P1912, DOI 10.1093/BRAIN/AWN093 | 47 |
| SMITH AG, 2006, DIABETES CARE, V29, P1294, DOI 10.2337/DC06-0224 | 44 |
| PERKINS BA, 2001, DIABETES CARE, V24, P250, DOI 10.2337/DIACARE.24.2.250 | 43 |
| TESFAYE S, 1996, DIABETOLOGIA, V39, P1377, DOI 10.1007/S001250050586 | 42 |
| LAURIA G, 2010, EUR J NEUROL, V17, P903, DOI 10.1111/J.1468-1331.2010.03023.X | 41 |
| DYCK PJ, 1997, NEUROLOGY, V49, P229, DOI 10.1212/WNL.49.1.229 | 39 |
| LAURIA G, 2010, J PERIPHER NERV SYST, V15, P202, DOI 10.1111/J.1529-8027.2010.00271.X | 39 |
| ABBOTT CA, 2002, DIABETIC MED, V19, P377, DOI 10.1046/J.1464-5491.2002.00698.X | 38 |
| ROLKE R, 2006, PAIN, V123, P231, DOI 10.1016/J.PAIN.2006.01.041 | 37 |
| BOULTON AJM, 2004, DIABETES CARE, V27, P1458, DOI 10.2337/DIACARE.27.6.1458 | 36 |
| PARTANEN J, 1995, NEW ENGL J MED, V333, P89, DOI 10.1056/NEJM199507133330203 | 36 |
| PHAM H, 2000, DIABETES CARE, V23, P606, DOI 10.2337/DIACARE.23.5.606 | 36 |
| SHUN CT, 2004, BRAIN, V127, P1593, DOI 10.1093/BRAIN/AWH180 | 36 |

### Source

Supplementary Table 14 Most Relevant Sources Top 25 ranked by number of articles. Of interest are Journal of Peripheral Neuropathy (20), next is Journal of Diabetes and it’s complications (14), finally the emergence of International Journal of Molecular Sciences (5).

| **Sources** | **Articles** |
| --- | --- |
| DIABETIC MEDICINE | 36 |
| DIABETES CARE | 35 |
| JOURNAL OF THE PERIPHERAL NERVOUS SYSTEM | 20 |
| NEUROLOGY | 19 |
| MUSCLE & NERVE | 17 |
| DIABETES RESEARCH AND CLINICAL PRACTICE | 15 |
| PAIN | 15 |
| JOURNAL OF DIABETES AND ITS COMPLICATIONS | 14 |
| DIABETOLOGIA | 12 |
| JOURNAL OF DIABETES INVESTIGATION | 10 |
| CURRENT OPINION IN NEUROLOGY | 9 |
| DIABETES-METABOLISM RESEARCH AND REVIEWS | 8 |
| BMJ OPEN DIABETES RESEARCH & CARE | 7 |
| JOURNAL OF NEUROLOGY NEUROSURGERY AND PSYCHIATRY | 7 |
| JOURNAL OF THE NEUROLOGICAL SCIENCES | 7 |
| PLOS ONE | 7 |
| CURRENT DIABETES REPORTS | 6 |
| FRONTIERS IN ENDOCRINOLOGY | 6 |
| JOURNAL OF THE AMERICAN PODIATRIC MEDICAL ASSOCIATION | 6 |
| ACTA DIABETOLOGICA | 5 |
| BRAIN | 5 |
| DIABETES & METABOLISM | 5 |
| EUROPEAN JOURNAL OF NEUROLOGY | 5 |
| EXPERIMENTAL AND CLINICAL ENDOCRINOLOGY & DIABETES | 5 |
| INTERNATIONAL JOURNAL OF MOLECULAR SCIENCES | 5 |

Supplementary Table 15 Top-25 Most Cited Sources. Diabetes car is clear leader in influencing practice across fields. Pain is becoming a major focus and source of insight.

| **Sources** | **Articles** |
| --- | --- |
| DIABETES CARE | 3015 |
| NEUROLOGY | 1557 |
| MUSCLE NERVE | 1153 |
| DIABETIC MED | 1095 |
| PAIN | 1006 |
| DIABETOLOGIA | 939 |
| DIABETES | 827 |
| BRAIN | 719 |
| J NEUROL NEUROSUR PS | 550 |
| ANN NEUROL | 521 |
| DIABETES RES CLIN PR | 399 |
| NEW ENGL J MED | 392 |
| J PERIPHER NERV SYST | 363 |
| J DIABETES COMPLICAT | 346 |
| J NEUROL SCI | 332 |
| J NEUROSCI | 283 |
| DIABETES-METAB RES | 266 |
| PLOS ONE | 266 |
| LANCET | 263 |
| ARCH NEUROL-CHICAGO | 228 |
| CLIN NEUROPHYSIOL | 227 |
| J NEUROL | 202 |
| P NATL ACAD SCI USA | 182 |
| EUR J NEUROL | 177 |
| EXP NEUROL | 157 |

Supplementary Table 16 Bradford’s Law Top 50 Sources. Each zone retains same cumulative frequency. First zone, core zone, disproportionate represents the highest performing sources and influence. Second zone, secondar zone, shows less impactful but still influential sources to the corpus.

| **SOURCE** | **Rank** | **Freq** | **cumFreq** | **Zone** |
| --- | --- | --- | --- | --- |
| DIABETIC MEDICINE | 1 | 36 | 36 | Zone 1 |
| DIABETES CARE | 2 | 35 | 71 | Zone 1 |
| JOURNAL OF THE PERIPHERAL NERVOUS SYSTEM | 3 | 20 | 91 | Zone 1 |
| NEUROLOGY | 4 | 19 | 110 | Zone 1 |
| MUSCLE & NERVE | 5 | 17 | 127 | Zone 1 |
| DIABETES RESEARCH AND CLINICAL PRACTICE | 6 | 15 | 142 | Zone 1 |
| PAIN | 7 | 15 | 157 | Zone 1 |
| JOURNAL OF DIABETES AND ITS COMPLICATIONS | 8 | 14 | 171 | Zone 1 |
| DIABETOLOGIA | 9 | 12 | 183 | Zone 1 |
| JOURNAL OF DIABETES INVESTIGATION | 10 | 10 | 193 | Zone 1 |
| CURRENT OPINION IN NEUROLOGY | 11 | 9 | 202 | Zone 1 |
| DIABETES-METABOLISM RESEARCH AND REVIEWS | 12 | 8 | 210 | Zone 2 |
| BMJ OPEN DIABETES RESEARCH & CARE | 13 | 7 | 217 | Zone 2 |
| JOURNAL OF NEUROLOGY NEUROSURGERY AND PSYCHIATRY | 14 | 7 | 224 | Zone 2 |
| JOURNAL OF THE NEUROLOGICAL SCIENCES | 15 | 7 | 231 | Zone 2 |
| PLOS ONE | 16 | 7 | 238 | Zone 2 |
| CURRENT DIABETES REPORTS | 17 | 6 | 244 | Zone 2 |
| FRONTIERS IN ENDOCRINOLOGY | 18 | 6 | 250 | Zone 2 |
| JOURNAL OF THE AMERICAN PODIATRIC MEDICAL ASSOCIATION | 19 | 6 | 256 | Zone 2 |
| ACTA DIABETOLOGICA | 20 | 5 | 261 | Zone 2 |
| BRAIN | 21 | 5 | 266 | Zone 2 |
| DIABETES & METABOLISM | 22 | 5 | 271 | Zone 2 |
| EUROPEAN JOURNAL OF NEUROLOGY | 23 | 5 | 276 | Zone 2 |
| EXPERIMENTAL AND CLINICAL ENDOCRINOLOGY & DIABETES | 24 | 5 | 281 | Zone 2 |
| INTERNATIONAL JOURNAL OF MOLECULAR SCIENCES | 25 | 5 | 286 | Zone 2 |
| PRIMARY CARE DIABETES | 26 | 5 | 291 | Zone 2 |
| ACTA NEUROLOGICA SCANDINAVICA | 27 | 4 | 295 | Zone 2 |
| CLINICAL NEUROPHYSIOLOGY | 28 | 4 | 299 | Zone 2 |
| JOURNAL OF CLINICAL NEUROPHYSIOLOGY | 29 | 4 | 303 | Zone 2 |
| JOURNAL OF DIABETES RESEARCH | 30 | 4 | 307 | Zone 2 |
| NEUROSCIENCE LETTERS | 31 | 4 | 311 | Zone 2 |
| SCIENTIFIC REPORTS | 32 | 4 | 315 | Zone 2 |
| ANTIOXIDANTS & REDOX SIGNALING | 33 | 3 | 318 | Zone 2 |
| CLINICAL THERAPEUTICS | 34 | 3 | 321 | Zone 2 |
| DIABETES | 35 | 3 | 324 | Zone 2 |
| DIABETES & METABOLISM JOURNAL | 36 | 3 | 327 | Zone 2 |
| DIABETES REVIEWS | 37 | 3 | 330 | Zone 2 |
| DIABETES TECHNOLOGY & THERAPEUTICS | 38 | 3 | 333 | Zone 2 |
| DIABETES THERAPY | 39 | 3 | 336 | Zone 2 |
| EUROPEAN JOURNAL OF PAIN | 40 | 3 | 339 | Zone 2 |
| EUROPEAN NEUROLOGY | 41 | 3 | 342 | Zone 2 |
| INTERNATIONAL WOUND JOURNAL | 42 | 3 | 345 | Zone 2 |
| JOURNAL OF CLINICAL AND DIAGNOSTIC RESEARCH | 43 | 3 | 348 | Zone 2 |
| JOURNAL OF CLINICAL MEDICINE | 44 | 3 | 351 | Zone 2 |
| JOURNAL OF DIABETES AND METABOLIC DISORDERS | 45 | 3 | 354 | Zone 2 |
| JOURNAL OF DIABETES SCIENCE AND TECHNOLOGY | 46 | 3 | 357 | Zone 2 |
| JOURNAL OF EVOLUTION OF MEDICAL AND DENTAL SCIENCES-JEMDS | 47 | 3 | 360 | Zone 2 |
| NEUROLOGIC CLINICS | 48 | 3 | 363 | Zone 2 |
| NOROPSIKIYATRI ARSIVI-ARCHIVES OF NEUROPSYCHIATRY | 49 | 3 | 366 | Zone 2 |
| PEDIATRIC DIABETES | 50 | 3 | 369 | Zone 2 |

### Countries

Supplementary Table 17 Top-25 Countries ranked using articles across period. Single Country Production (SCP) and Multiple Country Production (MCP) provide snapshot of collaboration ratio.

| **Country** | **Articles** | **SCP** | **MCP** | **Freq** | **MCP_Ratio** |
| --- | --- | --- | --- | --- | --- |
| USA | 126 | 101 | 25 | 0.214 | 0.198 |
| UNITED KINGDOM | 71 | 44 | 27 | 0.121 | 0.38 |
| **UNCLASSIFIED** | 32 | 30 | 2 | 0.054 | 0.063 |
| GERMANY | 30 | 17 | 13 | 0.051 | 0.433 |
| CHINA | 29 | 26 | 3 | 0.049 | 0.103 |
| ITALY | 27 | 18 | 9 | 0.046 | 0.333 |
| INDIA | 24 | 24 | 0 | 0.041 | 0 |
| CANADA | 22 | 16 | 6 | 0.037 | 0.273 |
| DENMARK | 19 | 12 | 7 | 0.032 | 0.368 |
| FRANCE | 19 | 16 | 3 | 0.032 | 0.158 |
| NETHERLANDS | 18 | 12 | 6 | 0.031 | 0.333 |
| AUSTRALIA | 17 | 11 | 6 | 0.029 | 0.353 |
| GREECE | 16 | 13 | 3 | 0.027 | 0.188 |
| JAPAN | 14 | 13 | 1 | 0.024 | 0.071 |
| TURKEY | 14 | 14 | 0 | 0.024 | 0 |
| SPAIN | 10 | 8 | 2 | 0.017 | 0.2 |
| CZECH REPUBLIC | 8 | 3 | 5 | 0.014 | 0.625 |
| QATAR | 8 | 1 | 7 | 0.014 | 0.875 |
| KOREA | 7 | 6 | 1 | 0.012 | 0.143 |
| IRAN | 5 | 5 | 0 | 0.008 | 0 |
| SINGAPORE | 5 | 5 | 0 | 0.008 | 0 |
| SWEDEN | 5 | 2 | 3 | 0.008 | 0.6 |
| BRAZIL | 4 | 4 | 0 | 0.007 | 0 |
| MALAYSIA | 4 | 0 | 4 | 0.007 | 1 |
| POLAND | 4 | 4 | 0 | 0.007 | 0 |

Supplementary Table 18 Top-10 Countries ranked using Single Country Production (SCP) across period. Rank shows SCP output.

| **Country** | **Articles** | **SCP** | **MCP** | **Freq** | **MCP_Ratio** |
| --- | --- | --- | --- | --- | --- |
| USA | 126 | 101 | 25 | 0.214 | 0.198 |
| UNITED KINGDOM | 71 | 44 | 27 | 0.121 | 0.38 |
| **UNCLASSIFIED** | 32 | 30 | 2 | 0.054 | 0.063 |
| CHINA | 29 | 26 | 3 | 0.049 | 0.103 |
| INDIA | 24 | 24 | 0 | 0.041 | 0 |
| ITALY | 27 | 18 | 9 | 0.046 | 0.333 |
| GERMANY | 30 | 17 | 13 | 0.051 | 0.433 |
| CANADA | 22 | 16 | 6 | 0.037 | 0.273 |
| FRANCE | 19 | 16 | 3 | 0.032 | 0.158 |
| TURKEY | 14 | 14 | 0 | 0.024 | 0 |

Supplementary Table 19 Top-10 Countries ranked using Multiple Country Production (MCP) across period. Rank shows MCP output.

| **Country** | **Articles** | **SCP** | **MCP** | **Freq** | **MCP_Ratio** |
| --- | --- | --- | --- | --- | --- |
| UNITED KINGDOM | 71 | 44 | 27 | 0.121 | 0.38 |
| USA | 126 | 101 | 25 | 0.214 | 0.198 |
| GERMANY | 30 | 17 | 13 | 0.051 | 0.433 |
| ITALY | 27 | 18 | 9 | 0.046 | 0.333 |
| DENMARK | 19 | 12 | 7 | 0.032 | 0.368 |
| QATAR | 8 | 1 | 7 | 0.014 | 0.875 |
| CANADA | 22 | 16 | 6 | 0.037 | 0.273 |
| NETHERLANDS | 18 | 12 | 6 | 0.031 | 0.333 |
| AUSTRALIA | 17 | 11 | 6 | 0.029 | 0.353 |
| CZECH REPUBLIC | 8 | 3 | 5 | 0.014 | 0.625 |

Supplementary Table 20 Top-25 Countries ranked using total citations

| **Country** | **TC** | **Average Article Citations** |
| --- | --- | --- |
| USA | 11735 | 93.10 |
| UNITED KINGDOM | 3892 | 54.80 |
| ITALY | 2681 | 99.30 |
| CANADA | 1361 | 61.90 |
| GERMANY | 1330 | 44.30 |
| NETHERLANDS | 921 | 51.20 |
| CHINA | 659 | 22.70 |
| FRANCE | 659 | 34.70 |
| AUSTRALIA | 511 | 30.10 |
| DENMARK | 451 | 23.70 |
| GREECE | 438 | 27.40 |
| SINGAPORE | 387 | 77.40 |
| INDIA | 361 | 15.00 |
| JAPAN | 343 | 24.50 |
| CZECH REPUBLIC | 316 | 39.50 |
| TURKEY | 271 | 19.40 |
| SPAIN | 238 | 23.80 |
| QATAR | 211 | 26.40 |
| ISRAEL | 173 | 57.70 |
| FINLAND | 125 | 41.70 |
| AUSTRIA | 99 | 33.00 |
| BAHRAIN | 99 | 33.00 |
| IRAN | 97 | 19.40 |
| SWITZERLAND | 90 | 45.00 |
| U ARAB EMIRATES | 86 | 43.00 |

Supplementary Table 21 Top-10 Countries ranked using average citations

| **Country** | **TC** | **Average Article Citations** |
| --- | --- | --- |
| ITALY | 2681 | 99.30 |
| USA | 11735 | 93.10 |
| SINGAPORE | 387 | 77.40 |
| CANADA | 1361 | 61.90 |
| ISRAEL | 173 | 57.70 |
| UNITED KINGDOM | 3892 | 54.80 |
| NETHERLANDS | 921 | 51.20 |
| SWITZERLAND | 90 | 45.00 |
| GERMANY | 1330 | 44.30 |
| U ARAB EMIRATES | 86 | 43.00 |

### Institutions

Supplementary Table 22 Top 20 affiliations of authors. Frequency of affiliation refers to the sum of authors times their institution. For The University of Manchester, across all papers from 1993-2023, shows 75 counts of authors associated to this institution. This helps identify research hubs for relevant research.

| **Affiliation** | **Frequency of Affiliation** |
| --- | --- |
| UNIVERSITY OF MANCHESTER | 75 |
| HARVARD UNIVERSITY | 44 |
| AALBORG UNIVERSITY | 41 |
| JOHNS HOPKINS UNIVERSITY | 37 |
| AARHUS UNIVERSITY | 35 |
| IRCCS ISTITUTO NEUROLOGICO BESTA | 34 |
| UNIVERSITY OF MICHIGAN | 32 |
| UNIVERSITY OF MICHIGAN SYSTEM | 32 |
| QATAR FOUNDATION (QF) | 30 |
| WEILL CORNELL MEDICAL COLLEGE QATAR | 30 |
| MAYO CLINIC | 29 |
| RUPRECHT KARLS UNIVERSITY HEIDELBERG | 28 |
| AALBORG UNIVERSITY HOSPITAL | 25 |
| HARVARD MEDICAL SCHOOL | 25 |
| HEINRICH HEINE UNIVERSITY DUSSELDORF | 25 |
| UNIVERSITY OF LONDON | 24 |
| UDICE-FRENCH RESEARCH UNIVERSITIES | 23 |
| UNIVERSITY OF LIVERPOOL | 22 |
| UNIVERSITY OF TORONTO | 22 |
| UNIVERSITY OF WASHINGTON | 22 |
