# Supplementary material for: Bibliometric Analysis: Insights Into the Podiatric Medicine Landscape of Diabetic Sensory Peripheral Neuropathy and Genomics
Source: J Foot Ankle Res. 2025 Jul 24;18(3):e70062. doi: 10.1002/jfa2.70062 (PMC12289441; doi:10.1002/jfa2.70062)
Supplement: Supplementary file 5 — Supporting Information S5 [file JFA2-18-e70062-s008.docx]

# Supplementary File 8 Influential Documents

Division of tranches reflected the spread of the papers. Each tranche was selected by the logical and visual concentration of papers.

Supplementary Table 24 Core papers across 1991-2015 (determined by performance metrics and Historiography) showing focus within tranche 1-3. Synopsis overview: First row highlights lead author, year, and journal. Second row themes and focus (with period open and closure alongside key trials, tools, or studies). Columns divide 1991-2015 tranches were work concentrated. Total period 1991-2023

|  | **Tranche 1; 1991-1998** | **Tranche 2; 2001-2005** | **Tranche 3; 2008-2015** |
| --- | --- | --- | --- |
| **Author, Year (Journal)** | Dyck et al^164^, 1993 (Neurology)  Young et al^40^, 1993 (Diabetologia)  Young et al^51^, 1994 (Diabetes Care)  Feldman et al^42^, 1994 (Diabetes Care)  Tesfaye et al^165^, 1996 (Diabetologia)  Adler et al^166^, 1997 (Diabetes Care)  McArthur et al^52^ 1998 (Neurology [Archive]) | Perkins et al^43^, 2001 (Diabetes Care)  Lacomis, D^62^, 2002 (Muscle and Nerve)  Meijer et al^7^, 2002, (Diabetic Medicine)  Shy et al^72^, 2003 (Neurology [American Academy])  Summer et al^44^, 2003 (Neurology)  Shun et al^53^, 2004 (Brain)  Boulton et al^167^, 2004 (Diabetes Care)  Gregg et al^56^, 2004 (Diabetes Care)  Boulton et al^70^, 2005 (Diabetes Care) | Devigili et al^9^, 2008 (Brain)  Lauria et al^71^, 2010 (European Journal of Neurology/Peripheral Nerve Society)  Tesfaye et al^10^, 2010 (Diabetes Care)  Lauria et al^168^, 2010 (Peripheral Nerve Society)  Backonja et al^169^, 2013 (Pain)  Azmi et al^66^, 2015 (Diabetes Care) |
| **Themes/Focus** | **Period opened with:**  Major prospective, cross-sectional studies withing diabetic cohort of type 1 and 2  **Focus:**  Improving assessments and diagnosis; vibratory and nerve conduction favoured.  Driven by clinical need/approach.  Period of high volume of primary data generation and tool/assessment generation.  Advice that tools are to assist diagnostics but not reliable to be fully diagnostic.  Predominantly USA/UK centric with European emergence.  Neurology and clinical diabetes driven.  **Major Trials:**  The Rochester Diabetic Neuropathy Study  The UK Prevalence Study 1993  Michigan Neuropathy Screening Instrument (MNSI) Study  EURODIAB IDDM Complications Study  Seattle Prospective Diabetic Foot Study  **Period closed with:**  Epidermal tissues investigation.  Early stages of objective assessment.  Normative reference ranges being sought. | **Period opened with:**  Evaluation and verification of new approaches.  Smaller cohort studies.  Emergence of pathological understanding.  **Focus:**  Improving assessments and diagnosis; increasing development/refinement of scoring systems.  Driven by clinical need/approach.  Seeking of parsimonious approaches e.g., Monofilament testing, to improve uptake and assessment.  Refinement of definitions and attempts to reclassify clinical scoring.  Quantitative Sensory Testing emerging as preferred, but period notes inadequate high-quality evidence.  Diabetes duration emerges as associated risk factor.  Increased literature using basic science to improve pathological underpinning.  Increase in European and East-Asian publishing.  Neuroscience and clinical diabetes driven with emerging rehabilitation.  **Major Trials/Studies:**  National Health and Nutrition Examination Survey.  The Diabetic Neuropathy Symptom Score.  **Period closed with:**  Expertise Statements and Review commentaries were high.  Consensus driven classifications of somatic neuropathies.  Increasing realisation of reliance upon non-reproducible studies.  Provision of practical guidelines | **Period opened with:**  Cohort studies investigating invasive and non-invasive testing.  Skin biopsy ‘gold standard’ recommendations and guidance.  **Focus:**  Increasing use of skin biopsy with normative ranges consolidated for clinical practice.  Emergence of expertise clusters globally with internationally establish study group.  Reliance upon reviews and consensus research to drive thinking, classification, definitions, and diagnosis.  Increased delineation of evidence supporting small-fibre neuropathy with pain and sensory neuropathy affecting large-fibre.  Limitations emerging on skin biopsy approach towards latter end of period; whilst reliable, has logistical challenges e.g., appropriate lab processing,  Emergence of Middle-East research.  Neurophysiology, Neuroscience, and clinical diabetes driving research.  **Major Trials/Studies:**  NeuPSIG Consensus  **Period closed with:**  Corneal Confocal Microscopy (CCM) contenting with peripheral nerve assessments in the lower for diagnostic potential and value.  Increase objective assessments in approach; small cohort numbers to provide meaning; emergence overlaps with small-fibre neuropathy. |

Supplementary Table 25 Core papers across 2016-2023 (determined by performance metrics and Historiography) showing focus within tranche 1-3. Synopsis overview: First row highlights lead author, year, and journal. Second row themes and focus (with period open and closure alongside key trials, tools, or studies). Columns divide 1991-2015 tranches were work concentrated. Total period 1991-2023.

|  | **Tranche 4; 2016-2019** | **Tranche 5; 2020-2023** |
| --- | --- | --- |
| Author, Year (Journal) | Themistocleous et al^170^, 2016 (Pain)  Pop-Busui et al^69^, 2017 (Diabetes Care)  Iqbal et al^57^, 2018 (Clinical therapeutics)  Sopacua et al^59^, 2018 (Peripheral Nerve Society)  Zaharia et al^171^, 2019 (Lancet) | Rosenberger et al^60^, 2020 (Neural Transmission)  Røiker et al^64^, 2020 (Diabetic Medicine)  Burgess et al^172^, 2021 (Diagnostic; MDPI)  Røiker et al^65^, 2021 (Diabetic Medicine)  McCray et al^173^, 2021 (Nature Communications)  Ziegler et al^58^, 2022 (Diabetes Research and Clinical Practice)  Ślęczkowska et al^174^, 2022 (Molecular Sciences)  Themistocleous et al^55^, 2023 (Brain Communications)  Røiker et al^175^, 2023 (Acta Diabetologica)  Røiker et al^176^, 2023 (Diabetes Science and Technology)  Røiker et al^49^, 2023 (Pain)  Croosu et al^177^, 2023 (Diabetes Care) |
| Themes/Focus | **Period opened with**  Smaller cohort students with deeper focus upon molecular medicine.  Attraction of UK research ‘powerhouses’ with sensory phenotyping being principle focus  **Focus:**  Painful neuropathy become prominent focus across clinical and neurosciences.  Recognition diabetic neuropathy is a diagnosis of exclusion.  Refinement in diabetes diagnosis and challenges of ‘pre-diabetes’.  Guidance towards prevention with emphasise of glycaemic management to protect nervous system.  Importance of early screening.  Recognition evidence fails to attain high robustness e.g., RCT, and reproducibility.  Recognition clinical presentation is late-stage diagnosis restricting prevention.  Channelopathies and genetic association linked to SFN; variants of interest emerging and their impact upon peripheral nervous system.  Period of increasing primary data generation from basic science (DNA sequencing)  Neuroscience, genetics/genomics, chemistry, physiology, clinical epidemiology, and diabetes driven.  **Major Trials:**  The German Diabetes Study Group  **Period closed with:**  Large cohort study and clinical trial.  Successful clustering of participants to diabetes types/sub-types using whole-body/adipose-tissue insulin resistance using extensive phenotyping. | **Period opened with:**  Global pandemic Covid_19.  Attraction towards phenotype-based stratification of neuropathy.  Review of state-of-the-art approaches personalised pain management.  Translation research priorities.  **Focus:**  Smaller cohort studies with targeted focus on mechanism or biomarker associations  Retrospective study increase; epidemiology focus  Painful neuropathies increased in prominence.  Recognition of Monofilament being appropriate only for advanced diabetic complication progression monitoring.  Neuronal excitability (particularly for pain) and pre-clinical presentation (sensory phenotype) are of increasing interest.  Vibratory threshold perception (VTP) re-entered corpus more directly with QTD  Importance of early screening.  Point-of-care testing emergence in literature.  Delphi process to assess consensus internationally employed, non-consensus evident across elements of screening, diagnosis, and management of diabetic sensorimotor polyneuropathy.  Reduced interested in large fibre assessment as preference for pain management stratification has higher focus.  Clinical medicine and endocrinology, Pain Medicine, Health Science and Technology, Nutrition and translational metabolism research, Diabetology, Biochemistry, Genetics/Genomics (clinical and research), Neurophysiology/plasticity, Epidemiology, and Neurology driven.  Toxicogenomic, Neuroscience and Regeneration research, Systems medicine Mitochondria research, Biomedical, Radiology, Anaesthesiology, and Orthopaedics emerging influencer.  **Major Trials:**  Methods for early detection of diabetic peripheral neuropathy (MEDON)  **Period closed with:**  Aalborg, Denmark significant contemporaneous contributor to corpus with focus upon translational medicine and epidemiology.  Germany, Netherlands, UK, and USA are major contributors to genetics/genomics and proteomics pathophysiology and aetiology.  Large public and genetic data/tissue repositories mainstreaming scientific analysis at-scale. |
