# Supplementary material for: Bibliometric Analysis: Insights Into the Podiatric Medicine Landscape of Diabetic Sensory Peripheral Neuropathy and Genomics
Source: J Foot Ankle Res. 2025 Jul 24;18(3):e70062. doi: 10.1002/jfa2.70062 (PMC12289441; doi:10.1002/jfa2.70062)
Supplement: Supplementary file 6 — Supporting Information S6 [file JFA2-18-e70062-s010.docx]

# Supplementary File 9 Intellectual and Conceptual Structures

See Supplementary File: Parameters (Network Analysis, Co-citation, and Co-word) for settings used in analysis.

## Historiography

Supplementary Table 26 Historiography data showing Paper, Title, Year, Local [LCS] and Global Citations [GCS]: Ranked top 20 by GCS. Colour row denotes batches visible from graphing [not shown] using citation frequency.

| **Paper** | **Title** | **Year** | **LCS** | **GCS** |
| --- | --- | --- | --- | --- |
| YOUNG MJ, 1993, DIABETOLOGIA DOI 10.1007/BF00400697 | A MULTICENTER STUDY OF THE PREVALENCE OF DIABETIC PERIPHERAL NEUROPATHY IN THE UNITED-KINGDOM HOSPITAL CLINIC POPULATION | 1993 | 110 | 1083 |
| DYCK PJ, 1993, NEUROLOGY DOI 10.1212/WNL.43.4.817 | THE PREVALENCE BY STAGED SEVERITY OF VARIOUS TYPES OF DIABETIC NEUROPATHY, RETINOPATHY, AND NEPHROPATHY IN A POPULATION-BASED COHORT -THE ROCHESTER DIABETIC NEUROPATHY STUDY | 1993 | 70 | 1047 |
| LAURIA G, 2010, EUR J NEUROL DOI 10.1111/J.1468-1331.2010.03023.X | EUROPEAN FEDERATION OF NEUROLOGICAL SOCIETIES/PERIPHERAL NERVE SOCIETY GUIDELINE ON THE USE OF SKIN BIOPSY IN THE DIAGNOSIS OF SMALL FIBER NEUROPATHY. REPORT OF A JOINT TASK FORCE OF THE EUROPEAN FE-DERATION OF NEUROLOGICAL SOCIETIES AND THE PERIPHERAL NERVE SOCIETY | 2010 | 41 | 584 |
| BOULTON AJM, 2004, DIABETES CARE DOI 10.2337/DIACARE.27.6.1458 | DIABETIC SOMATIC NEUROPATHIES | 2004 | 36 | 574 |
| SUMNER CJ, 2003, NEUROLOGY DOI 10.1212/WNL.60.1.108 | THE SPECTRUM OF NEUROPATHY IN DIABETES AND IMPAIRED GLUCOSE TOLERANCE | 2003 | 51 | 518 |
| DEVIGILI G, 2008, BRAIN DOI 10.1093/BRAIN/AWN093 | THE DIAGNOSTIC CRITERIA FOR SMALL FIBRE NEUROPATHY: FROM SYMPTOMS TO NEUROPATHOLOGY | 2008 | 47 | 510 |
| TESFAYE S, 1996, DIABETOLOGIA DOI 10.1007/S001250050586 | PREVALENCE OF DIABETIC PERIPHERAL NEUROPATHY AND ITS RELATION TO GLYCAEMIC CONTROL AND POTENTIAL RISK FACTORS: THE EURODIAB IDDM COMPLICATIONS STUDY | 1996 | 42 | 445 |
| YOUNG MJ, 1994, DIABETES CARE DOI 10.2337/DIACARE.17.6.557 | THE PREDICTION OF DIABETIC NEUROPATHIC FOOT ULCERATION USING VIBRATION PERCEPTION THRESHOLDS - A PROSPECTIVE-STUDY | 1994 | 83 | 428 |
| MCARTHUR JC, 1998, ARCH NEUROL-CHICAGO DOI 10.1001/ARCHNEUR.55.12.1513 | EPIDERMAL NERVE FIBER DENSITY - NORMATIVE REFERENCE RANGE AND DIAGNOSTIC EFFICIENCY | 1998 | 35 | 419 |
| LAURIA G, 2005, EUR J NEUROL DOI 10.1111/J.1468-1331.2005.01260.X | EFNS GUIDELINES ON THE USE OF SKIN BIOPSY IN THE DIAGNOSIS OF PERIPHERAL NEUROPATHY | 2005 | 25 | 394 |
| PERKINS BA, 2001, DIABETES CARE DOI 10.2337/DIACARE.24.2.250 | SIMPLE SCREENING TESTS FOR PERIPHERAL NEUROPATHY IN THE DIABETES CLINIC | 2001 | 43 | 376 |
| BACKONJA M, 2013, PAIN DOI 10.1016/J.PAIN.2013.05.047 | VALUE OF QUANTITATIVE SENSORY TESTING IN NEUROLOGICAL AND PAIN DISORDERS: NEUPSIG CONSENSUS | 2013 | 27 | 368 |
| SHY ME, 2003, NEUROLOGY DOI 10.1212/01.WNL.0000058546.16985.11 | QUANTITATIVE SENSORY TESTING - REPORT OF THE THERAPEUTICS AND TECHNOLOGY ASSESSMENT SUBCOMMITTEE OF THE AMERICAN ACADEMY OF NEUROLOGY | 2003 | 33 | 344 |
| LAURIA G, 2010, J PERIPHER NERV SYST DOI 10.1111/J.1529-8027.2010.00271.X | INTRAEPIDERMAL NERVE FIBER DENSITY AT THE DISTAL LEG: A WORLDWIDE NORMATIVE REFERENCE STUDY | 2010 | 39 | 317 |
| LACOMIS D, 2002, MUSCLE NERVE DOI 10.1002/MUS.10181 | SMALL-FIBER NEUROPATHY | 2002 | 28 | 277 |
| PERIQUET MI, 1999, NEUROLOGY DOI 10.1212/WNL.53.8.1641 | PAINFUL SENSORY NEUROPATHY - PROSPECTIVE EVALUATION USING SKIN BIOPSY | 1999 | 32 | 272 |
| SHUN CT, 2004, BRAIN DOI 10.1093/BRAIN/AWH180 | SKIN DENERVATION IN TYPE 2 DIABETES: CORRELATIONS WITH DIABETIC DURATION AND FUNCTIONAL IMPAIRMENTS | 2004 | 36 | 258 |
| ADLER AI, 1997, DIABETES CARE DOI 10.2337/DIACARE.20.7.1162 | RISK FACTORS FOR DIABETIC PERIPHERAL SENSORY NEUROPATHY - RESULTS OF THE SEATTLE PROSPECTIVE DIABETIC FOOT STUDY | 1997 | 27 | 210 |
| MEIJER JWG, 2002, DIABETIC MED DOI 10.1046/J.1464-5491.2002.00819.X | SYMPTOM SCORING SYSTEMS TO DIAGNOSE DISTAL POLYNEUROPATHY IN DIABETES: THE DIABETIC NEUROPATHY SYMPTOM SCORE | 2002 | 27 | 199 |
| SMITH AG, 2001, NEUROLOGY DOI 10.1212/WNL.57.9.1701 | EPIDERMAL NERVE INNERVATION IN IMPAIRED GLUCOSE TOLERANCE AND DIABETES-ASSOCIATED NEUROPATHY | 2001 | 27 | 186 |

Supplementary Table 27 Historiography data showing Paper, Title, Year, Local [LCS] and Global Citations [GCS]: Ranked top 20 by LCS. Colour row denotes batches visible from graphing [not shown] using citation frequency.

| **Paper** | **Title** | **Year** | **LCS** | **GCS** |
| --- | --- | --- | --- | --- |
| YOUNG MJ, 1993, DIABETOLOGIA DOI 10.1007/BF00400697 | A MULTICENTER STUDY OF THE PREVALENCE OF DIABETIC PERIPHERAL NEUROPATHY IN THE UNITED-KINGDOM HOSPITAL CLINIC POPULATION | 1993 | 110 | 1083 |
| YOUNG MJ, 1994, DIABETES CARE DOI 10.2337/DIACARE.17.6.557 | THE PREDICTION OF DIABETIC NEUROPATHIC FOOT ULCERATION USING VIBRATION PERCEPTION THRESHOLDS - A PROSPECTIVE-STUDY | 1994 | 83 | 428 |
| DYCK PJ, 1993, NEUROLOGY DOI 10.1212/WNL.43.4.817 | THE PREVALENCE BY STAGED SEVERITY OF VARIOUS TYPES OF DIABETIC NEUROPATHY, RETINOPATHY, AND NEPHROPATHY IN A POPULATION-BASED COHORT -THE ROCHESTER DIABETIC NEUROPATHY STUDY | 1993 | 70 | 1047 |
| SUMNER CJ, 2003, NEUROLOGY DOI 10.1212/WNL.60.1.108 | THE SPECTRUM OF NEUROPATHY IN DIABETES AND IMPAIRED GLUCOSE TOLERANCE | 2003 | 51 | 518 |
| DEVIGILI G, 2008, BRAIN DOI 10.1093/BRAIN/AWN093 | THE DIAGNOSTIC CRITERIA FOR SMALL FIBRE NEUROPATHY: FROM SYMPTOMS TO NEUROPATHOLOGY | 2008 | 47 | 510 |
| PERKINS BA, 2001, DIABETES CARE DOI 10.2337/DIACARE.24.2.250 | SIMPLE SCREENING TESTS FOR PERIPHERAL NEUROPATHY IN THE DIABETES CLINIC | 2001 | 43 | 376 |
| TESFAYE S, 1996, DIABETOLOGIA DOI 10.1007/S001250050586 | PREVALENCE OF DIABETIC PERIPHERAL NEUROPATHY AND ITS RELATION TO GLYCAEMIC CONTROL AND POTENTIAL RISK FACTORS: THE EURODIAB IDDM COMPLICATIONS STUDY | 1996 | 42 | 445 |
| LAURIA G, 2010, EUR J NEUROL DOI 10.1111/J.1468-1331.2010.03023.X | EUROPEAN FEDERATION OF NEUROLOGICAL SOCIETIES/PERIPHERAL NERVE SOCIETY GUIDELINE ON THE USE OF SKIN BIOPSY IN THE DIAGNOSIS OF SMALL FIBER NEUROPATHY. REPORT OF A JOINT TASK FORCE OF THE EUROPEAN FE-DERATION OF NEUROLOGICAL SOCIETIES AND THE PERIPHERAL NERVE SOCIETY | 2010 | 41 | 584 |
| LAURIA G, 2010, J PERIPHER NERV SYST DOI 10.1111/J.1529-8027.2010.00271.X | INTRAEPIDERMAL NERVE FIBER DENSITY AT THE DISTAL LEG: A WORLDWIDE NORMATIVE REFERENCE STUDY | 2010 | 39 | 317 |
| SHUN CT, 2004, BRAIN DOI 10.1093/BRAIN/AWH180 | SKIN DENERVATION IN TYPE 2 DIABETES: CORRELATIONS WITH DIABETIC DURATION AND FUNCTIONAL IMPAIRMENTS | 2004 | 36 | 258 |
| BOULTON AJM, 2004, DIABETES CARE DOI 10.2337/DIACARE.27.6.1458 | DIABETIC SOMATIC NEUROPATHIES | 2004 | 36 | 574 |
| MCARTHUR JC, 1998, ARCH NEUROL-CHICAGO DOI 10.1001/ARCHNEUR.55.12.1513 | EPIDERMAL NERVE FIBER DENSITY - NORMATIVE REFERENCE RANGE AND DIAGNOSTIC EFFICIENCY | 1998 | 35 | 419 |
| SHY ME, 2003, NEUROLOGY DOI 10.1212/01.WNL.0000058546.16985.11 | QUANTITATIVE SENSORY TESTING - REPORT OF THE THERAPEUTICS AND TECHNOLOGY ASSESSMENT SUBCOMMITTEE OF THE AMERICAN ACADEMY OF NEUROLOGY | 2003 | 33 | 344 |
| PERIQUET MI, 1999, NEUROLOGY DOI 10.1212/WNL.53.8.1641 | PAINFUL SENSORY NEUROPATHY - PROSPECTIVE EVALUATION USING SKIN BIOPSY | 1999 | 32 | 272 |
| LACOMIS D, 2002, MUSCLE NERVE DOI 10.1002/MUS.10181 | SMALL-FIBER NEUROPATHY | 2002 | 28 | 277 |
| ADLER AI, 1997, DIABETES CARE DOI 10.2337/DIACARE.20.7.1162 | RISK FACTORS FOR DIABETIC PERIPHERAL SENSORY NEUROPATHY - RESULTS OF THE SEATTLE PROSPECTIVE DIABETIC FOOT STUDY | 1997 | 27 | 210 |
| SMITH AG, 2001, NEUROLOGY DOI 10.1212/WNL.57.9.1701 | EPIDERMAL NERVE INNERVATION IN IMPAIRED GLUCOSE TOLERANCE AND DIABETES-ASSOCIATED NEUROPATHY | 2001 | 27 | 186 |
| MEIJER JWG, 2002, DIABETIC MED DOI 10.1046/J.1464-5491.2002.00819.X | SYMPTOM SCORING SYSTEMS TO DIAGNOSE DISTAL POLYNEUROPATHY IN DIABETES: THE DIABETIC NEUROPATHY SYMPTOM SCORE | 2002 | 27 | 199 |
| BACKONJA M, 2013, PAIN DOI 10.1016/J.PAIN.2013.05.047 | VALUE OF QUANTITATIVE SENSORY TESTING IN NEUROLOGICAL AND PAIN DISORDERS: NEUPSIG CONSENSUS | 2013 | 27 | 368 |
| LAURIA G, 2005, EUR J NEUROL DOI 10.1111/J.1468-1331.2005.01260.X | EFNS GUIDELINES ON THE USE OF SKIN BIOPSY IN THE DIAGNOSIS OF PERIPHERAL NEUROPATHY | 2005 | 25 | 394 |

## Network Analysis

## Co-citation


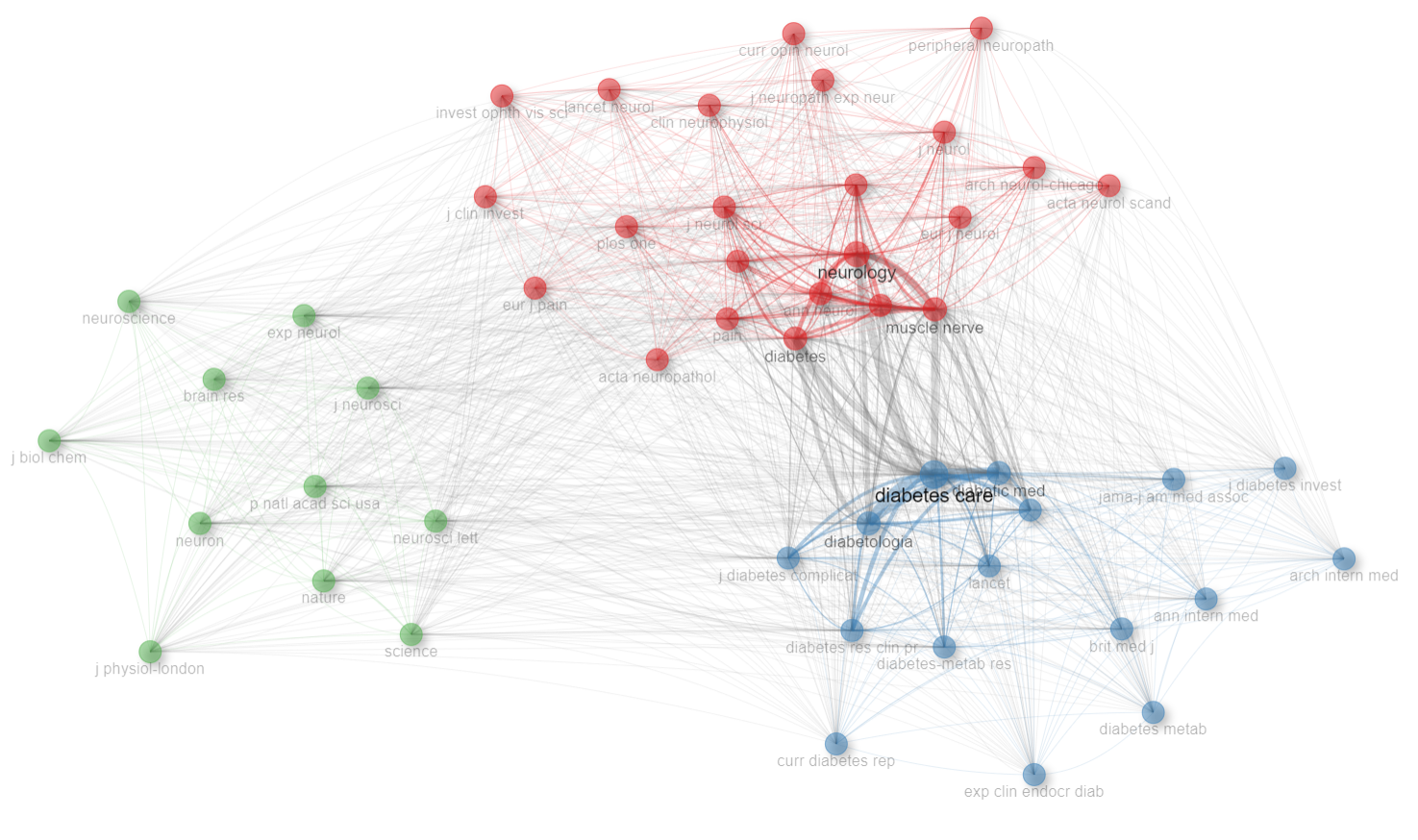


Supplementary Figure 5 Co-citation network analysis of journals using Fruchterman & Reingold layout. Three (3) distinct clusters emerge to show the shortest path to information exchanged. Thicker connections are indicative of the flow of information. Where the graphic becomes denser with connections, and journals proximity to one another, information exchange is highest i.e., most effective. Blue cluster centres on diabetes as a condition in care and management; Diabetes Care, Diabetic Medicine, Diabetologia share strongest connection within cluster and have closest proximity to red cluster versus Experimental Clinical Endocrinology and Diabetes Metabolism which are less co-cited and therefore, distant non-core information exchangers. Red cluster centre on clinical neurology; Neurology, Diabetes, and Muscle & Nerve are strongest connections within cluster and have closest proximity to aforementioned journal sources. Pain and peripheral neuropathy are distal comparatively. The green cluster centres loosely on neuroscience inclusive of biochemistry and physiology; no distinct clusters exist within.


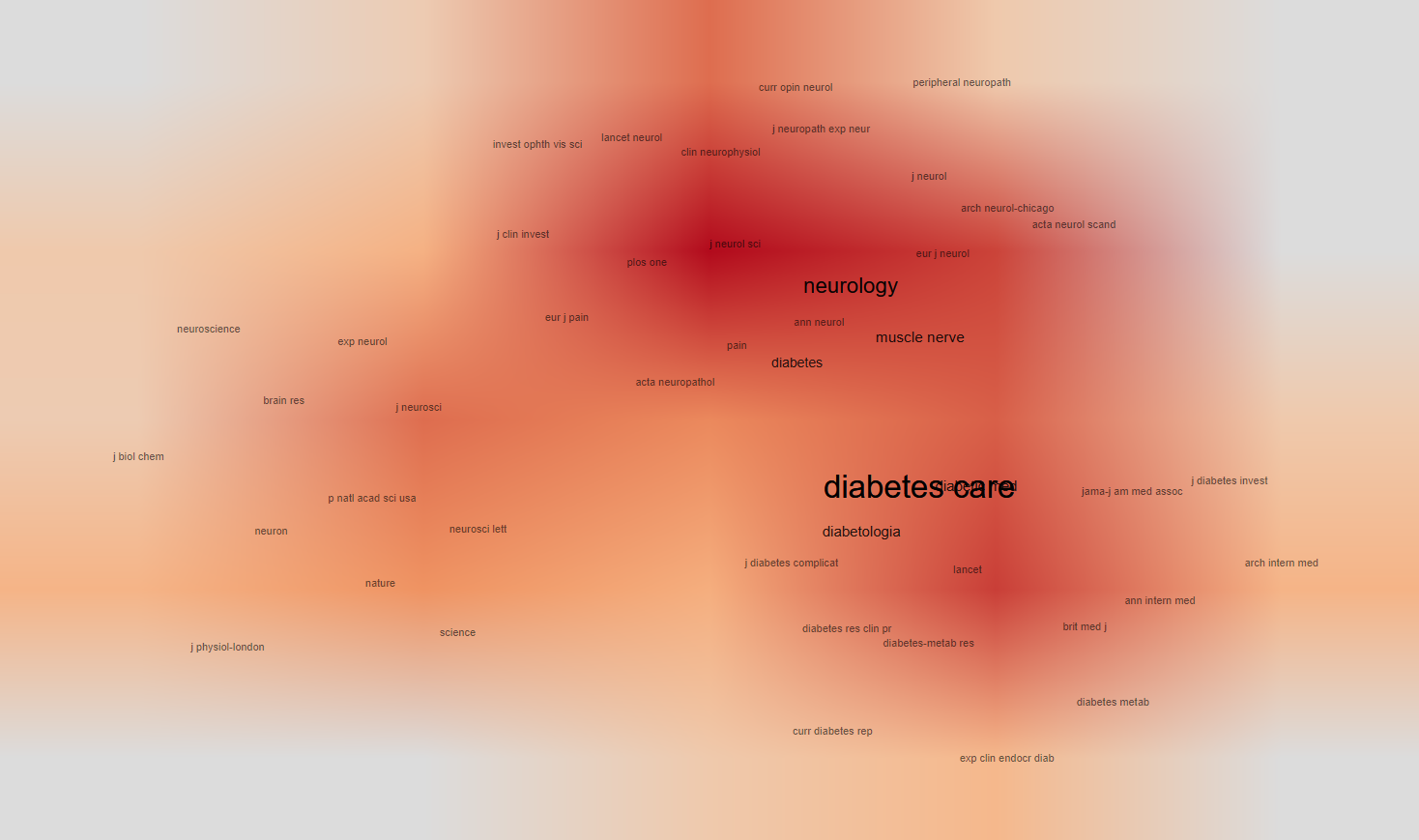


Supplementary Figure 6. Density plot of Fruchterman & Reingold layout co-citation network analysis of sources. Three (3) distinct densities (diabetes care | neurology, journal of neurological science | journal of neuroscience) match Supplementary Figure 5 to provide reader a reduction in complexity and highlight the concentration of structures in the data. Analogous to neighbourhoods, the eye is drawn to core zones readily. Deeper red indicates highest density (warmth) and lighter orange (cooler) is least dense. Higher density reflects increased co-citation of references.

## Co-word (Co-occurrence)


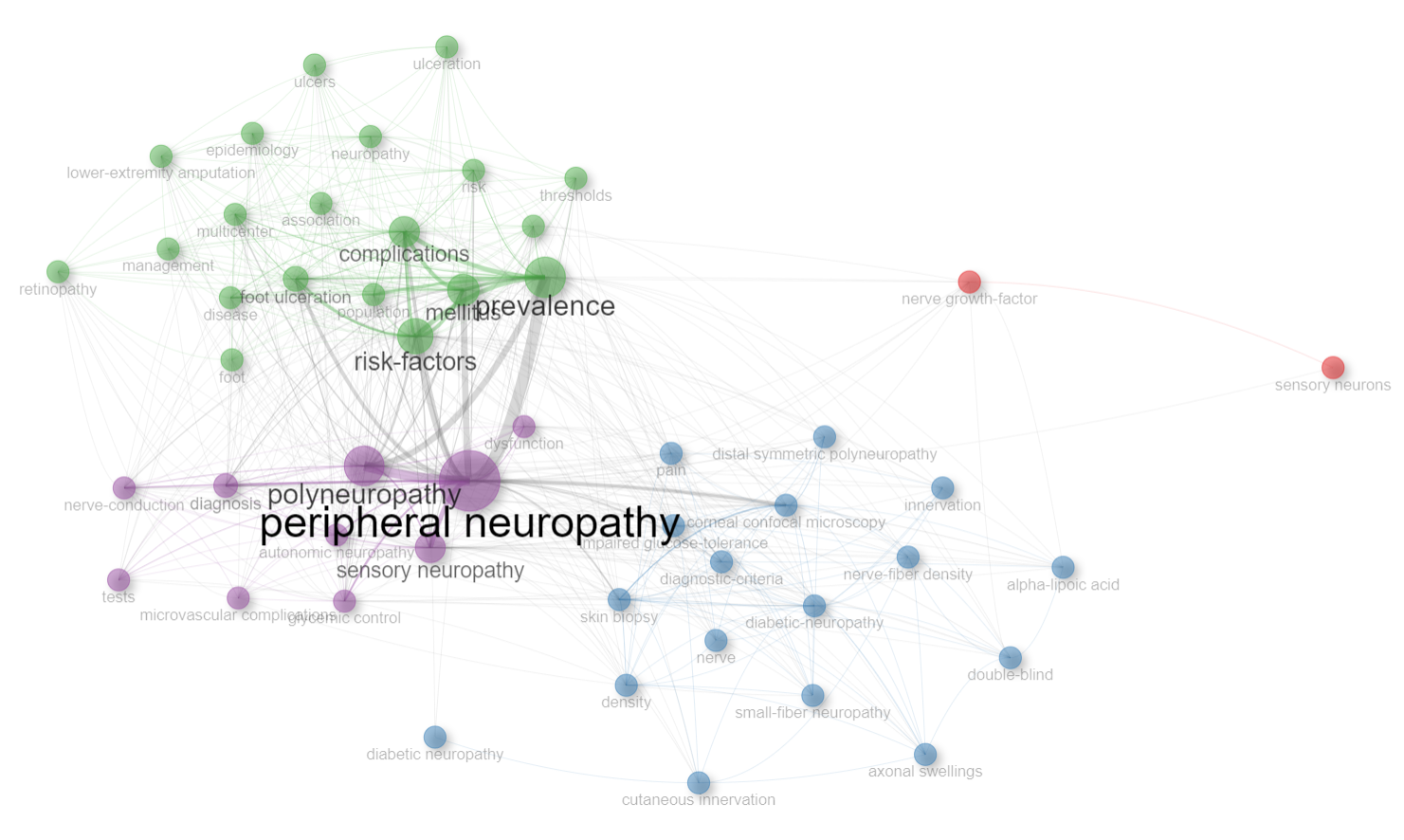


Supplementary Figure 7 Keyword Plus co-occurrence network analysis using Fruchterman & Reingold layout [**No filters applied**]. Four (4) distinct clusters emerge to show the shortest path to information exchanged. Thicker connections are indicative of the co-residing information (concepts/overlaps), Keywords/phrases have larger circles to reflect prominence, and their proximity reveals where highest co-occurrence occurs i.e., most accessible and understood (shared meaning). Purple cluster centres on neuropathic complications and pathophysiology; Peripheral Neuropathy, Polyneuropathy, and Sensory neuropathy share the strongest connections within cluster and have closest proximity to green cluster. **Prevalence, Risk-factors, Mellitus, Complications, and Foot ulcerations make up strongest connections within green cluster** centring around epidemiology terms; purple and green represent core concepts. Blue cluster represents the underlying biology and centres around neuroanatomy and diagnostics; corneal confocal microscopy and skin biopsy link to peripheral neuropathy in purple but within cluster; connections are sparse and evenly dispersed. Red cluster is loosely connected with nerve-growth factor and sensory neurons indicative of early concepts not yet fully formed.


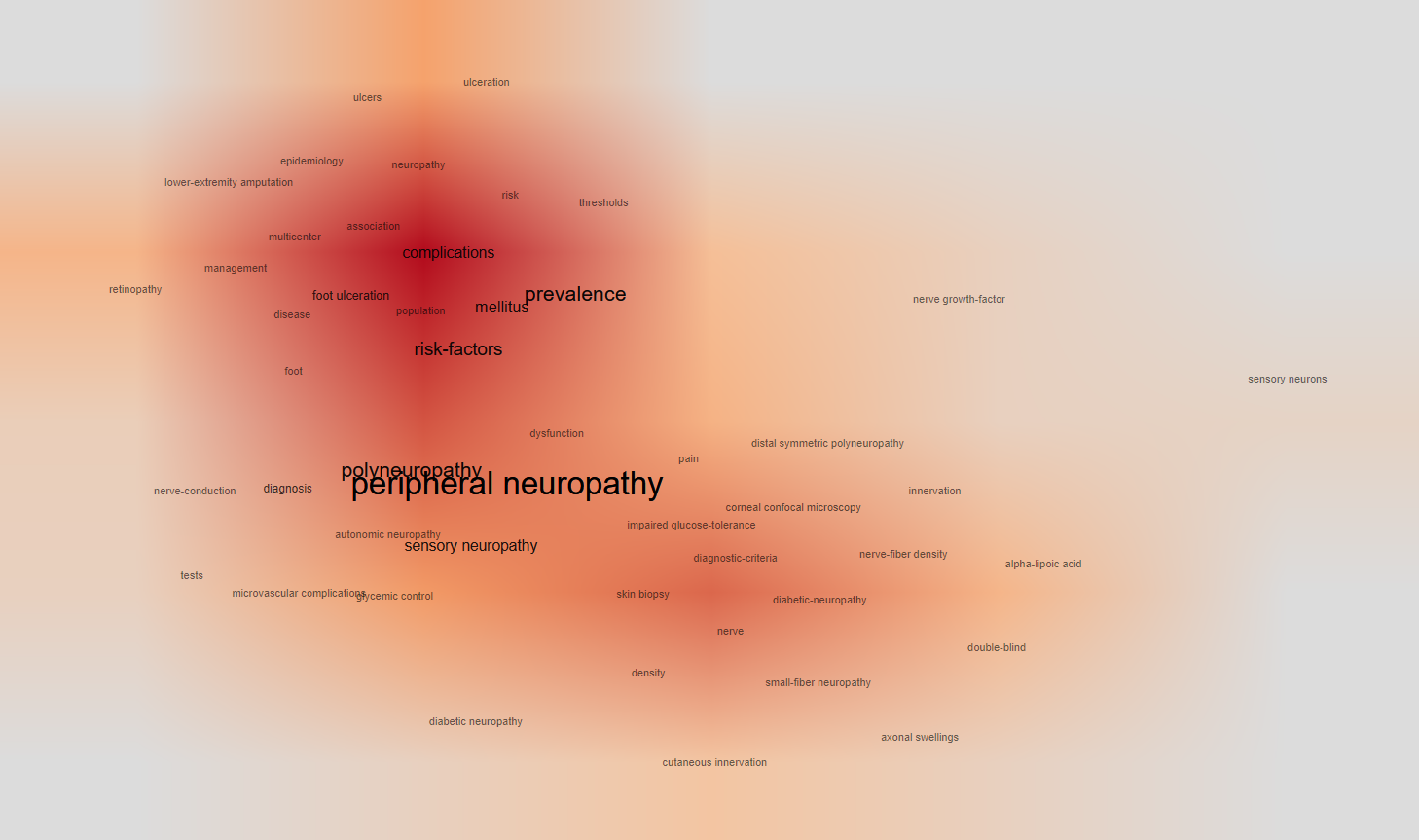


Supplementary Figure 8 Density plot of Fruchterman & Reingold layout of Keyword plus co-occurrence network analysis [No filters applied]. Three (3) distinct densities are visible (complications, risk-factors, prevalence|peripheral neuropathy, polyneurpahty|diagnostic critiera) and provide reader reduced complexity to see imporant conceptual structures in the data. An analogy to neighbourhoods can help the reader appreciate corpus when mapping; deeper red indicates highest density (warmth) and lighter orange (cooler) is least dense.


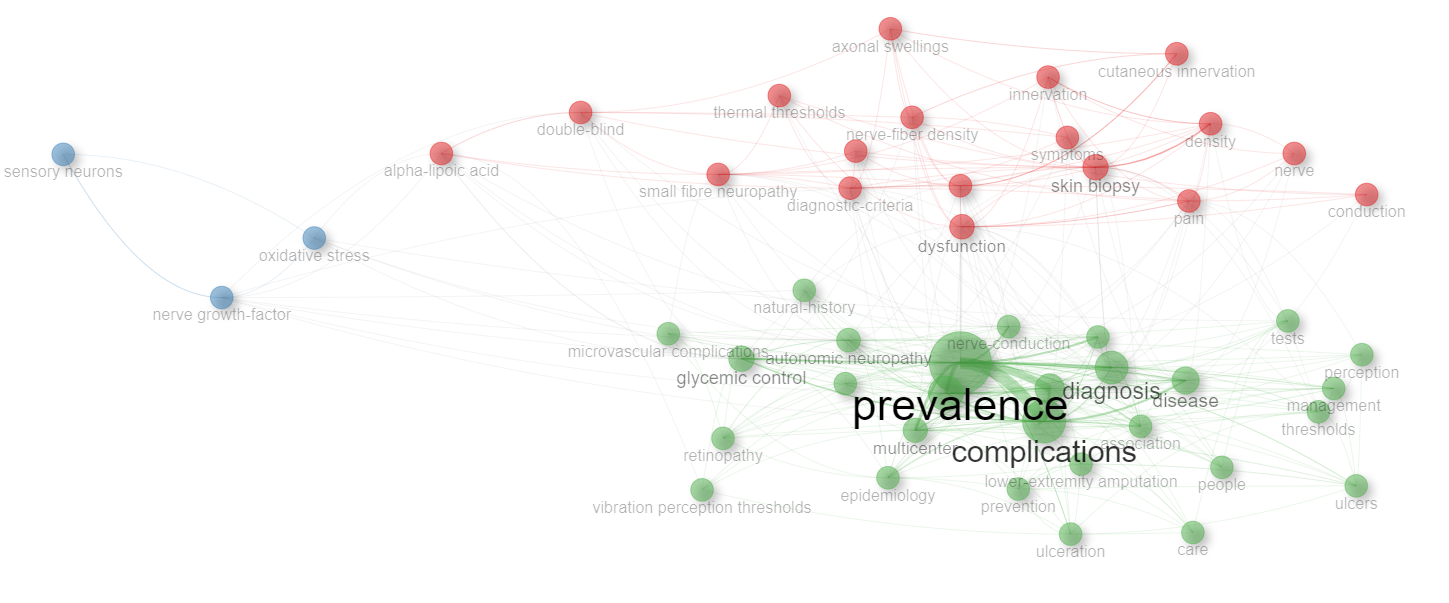


Supplementary Figure 9 Keyword plus co-occurrence network analysis using Fruchterman & Reingold layout [**Both filters applied**]. Filtering provided important re-clustering; groups dropped to reveal three (3) distinct clusters showing the shortest path to information exchanged. Previously fringe topics merge represented by blue cluster. Thicker connections are indicative of the co-residing information (concepts/overlaps), Keywords/phrases have larger circles to reflect prominence, and their proximity reveals where highest co-occurrence occurs i.e., most accessible and understood (shared meaning). Green cluster centres on neuropathic complications, pathophysiology, epidemiology, and assessment; Prevalence, complications, diagnosis, and population share strongest connection within cluster. Red cluster has weak links to green reflected by increased distance between clusters and ‘dysfunction’ showing the most prominent connection; red cluster centres around the underlying biology, neuroanatomy, and diagnostic-criteria. Blue cluster resides furthest from green’s core terms.


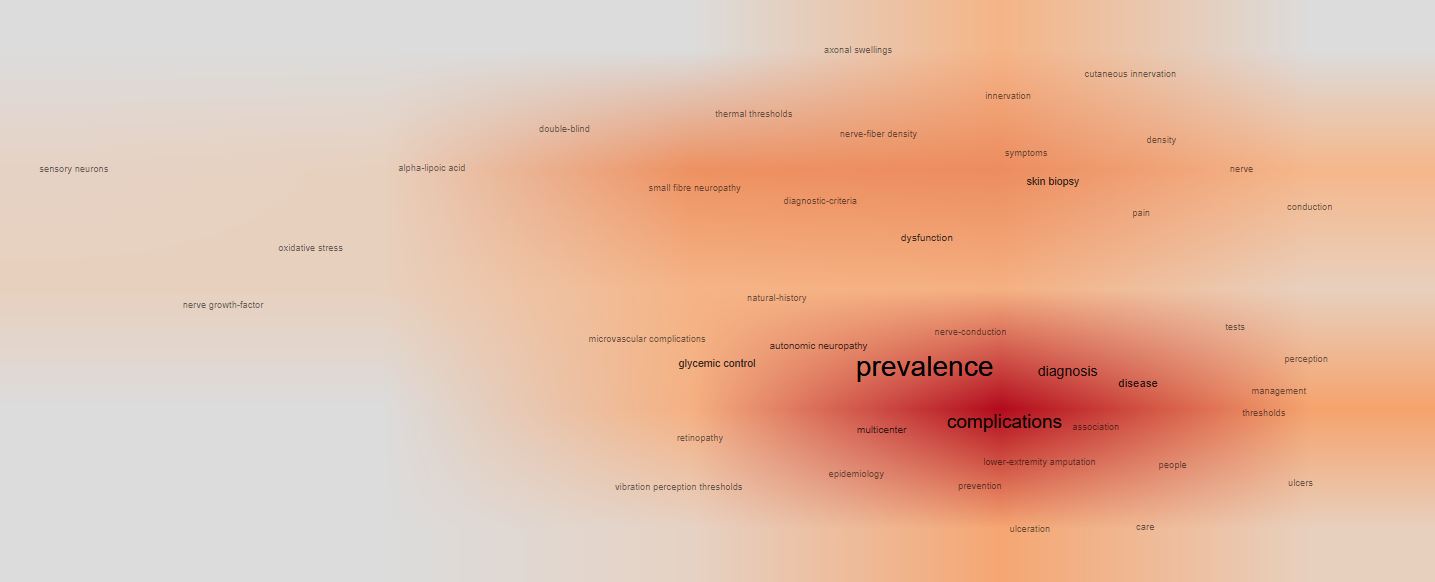


Supplementary Figure 10 Density plot of Fruchterman & Reingold layout of Keyword plus co-occurrence network analysis [**Both filters applied**]. One (1) distinct density neighbourhood appears (prevalence, complication, diagnosis) with smaller regions above (skin biopsy: small fibre neuropathy) that are visibly darker but lacking equivalent concentration. The deeper red indicates highest density (warmth) and lighter orange (cooler) is least dense. This figures highlights conceptual structures in the data, both formed (dark red) and forming (orange). Important to note filters reveal within the dataset new meaning and investigation is forming i.e., sensory neurons, comparative to established terms that represent the most pressing issues.


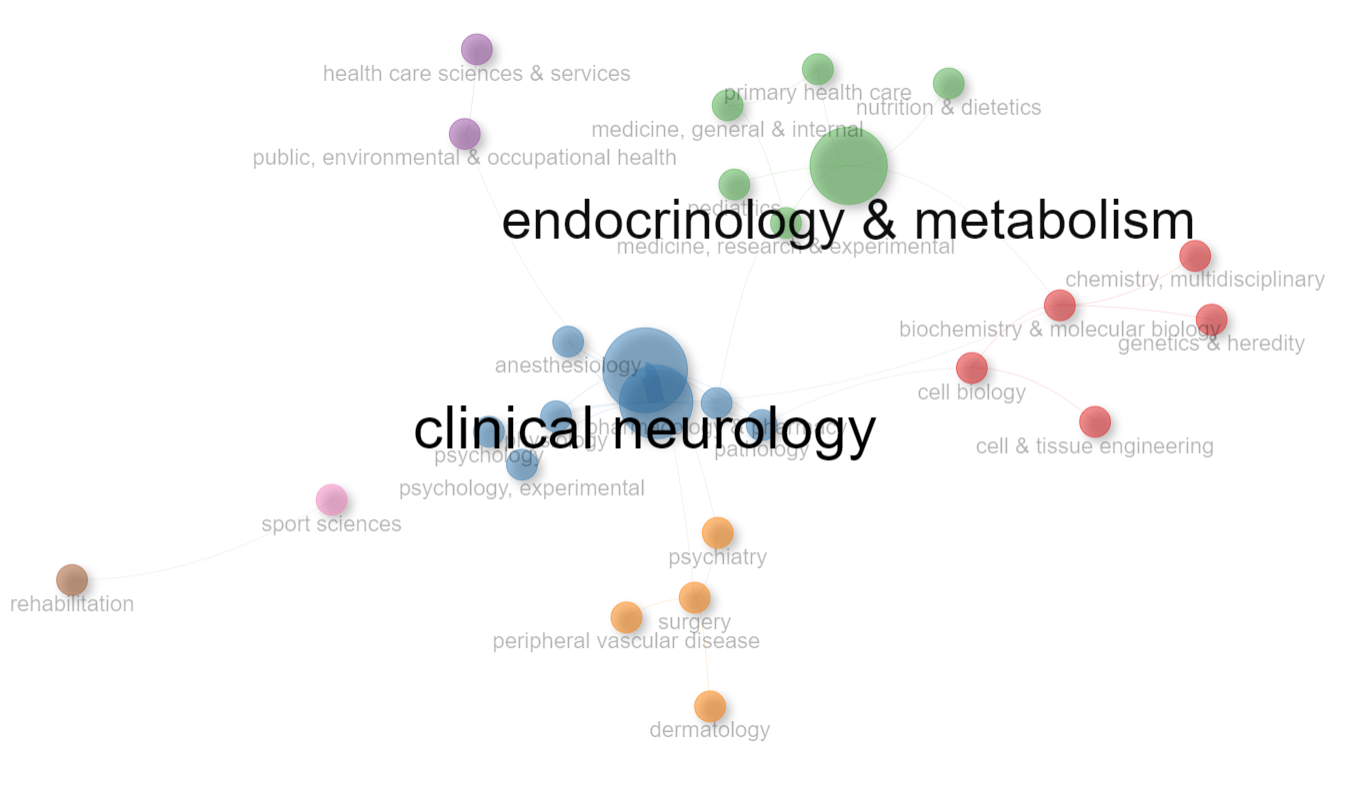


Supplementary Figure 11 Subject Categories (Web of Science) co-occurrence network analysis using Fruchterman & Reingold layout [**No filters applicable**]. Seven (7) distinct clusters emerge to show the shortest path to shared information exchanged. Subject categories are represented in size; larger equates to greater share of dataset concepts being within. Blues clusters was most prominent hosting neuroscience and clinical neurology with notable overlap; the periphery of this group held anaesthesia and psychology, experimental. Green cluster revealed endocrinology and metabolism as dominant host; primary health care and human& dietetics were more peripheral. Red cluster had evenly contributing categories centred on biochemistry & molecular biology, genetics & hereditary, and cell & tissue engineering. Orange had evenly contributing categories with surgery, psychiatry, peripheral vascular disease, and dermatology. Purple hosted health care science & services and public, environmental & occupational health; pink hosted sports science; and brown hosted rehabilitation. There were distinct and loosely connected to core subject category clusters.


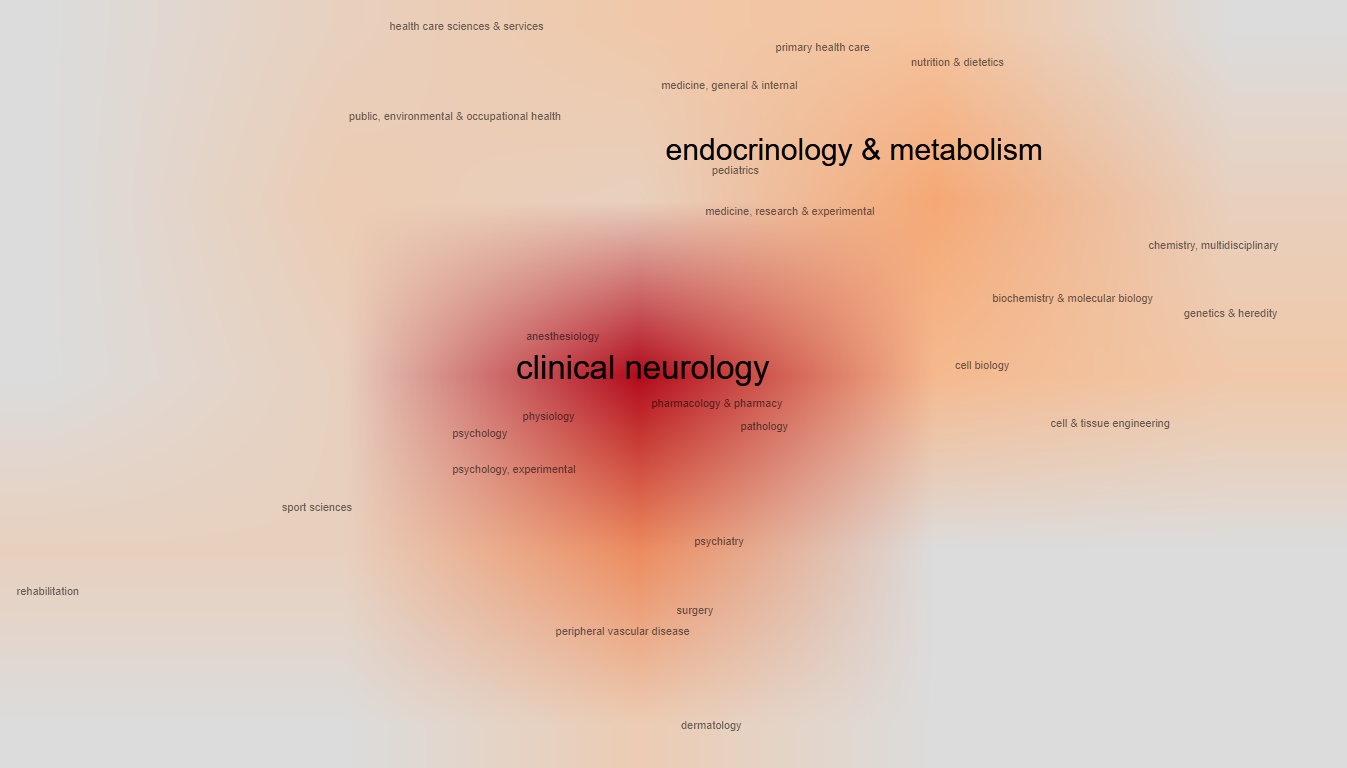


Supplementary Figure 12 Density plot of Fruchterman & Reingold layout of Subject Categories (Web of Science) co-occurrence network analysis [**No filters applicable**]. One (1) distinct density neighbourhood appears (clinical neurology) with smaller region above (endocrinology & metabolism) visible but lacking equivalent concentration. Deeper red indicates highest density (warmth) and lighter orange (cooler) is least dense. Primary care, health care science & service, and public, environmental & occupational health held least co-occurrence i.e., colder regions of interest. The intersection between metabolism, biochemistry & molecular biology, and clinical neurology suggest reveal darker orange indicating ‘warming’ area of interest.


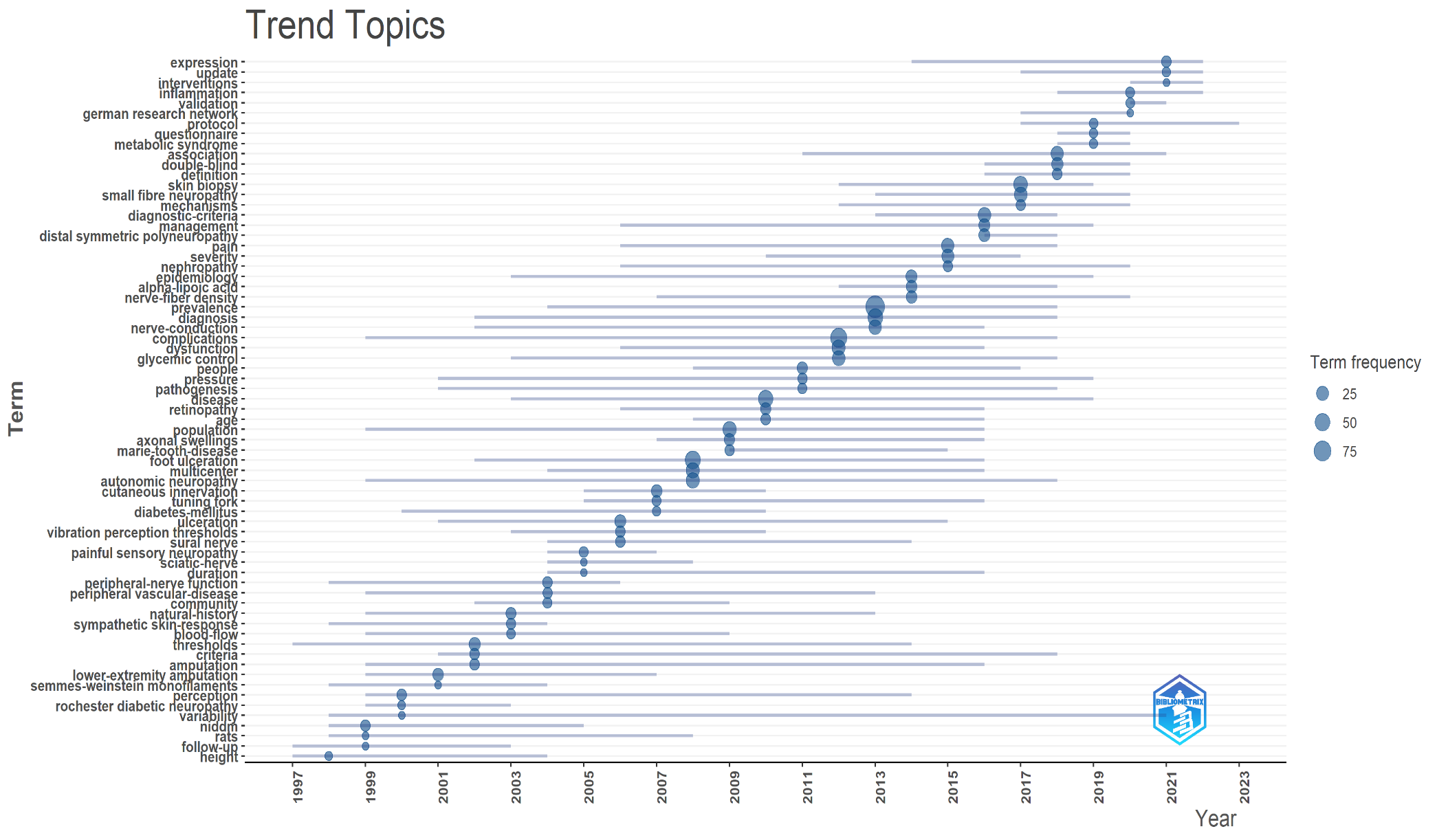


Supplementary Figure 13 Trend Topic breakdown; parameters frequency 5 word per year using 3 keyword plus. Across the years terms represented by bubbles show the highest performing concepts under investigation. Nerve Conduction as an assessment tool covers a large range and is the most contemporary clinical assessment tool peaking in usage ~2012 (n=27), however it is not readily available to podiatrists. The next actionable, point-of-care testing is the Vibration Perception Thresholds and Tuning Fork peaking ~2006/7 (n=12; n=9) respectively. As such, this became the focus of discussion as it was more contemporary than Semmes-Weinstein Monofilaments that peaked ~2001 (n=5).
