# Supplementary material for: Bibliometric Analysis: Insights Into the Podiatric Medicine Landscape of Diabetic Sensory Peripheral Neuropathy and Genomics
Source: J Foot Ankle Res. 2025 Jul 24;18(3):e70062. doi: 10.1002/jfa2.70062 (PMC12289441; doi:10.1002/jfa2.70062)
Supplement: Supplementary file 7 — Supporting Information S7 [file JFA2-18-e70062-s011.docx]

# Supplementary File 10 Parameters (Network Analysis, Co-citation, and Co-word)

Exclusion and synonyms filters applied to reduce non-relevance and duplication respectively.

## Web of Science Categories for Frequent Word

Subject Categories (WoS); 15 Words: Word occurrence by Square Root (*Selected to improve visual distribution and mitigate large, skewed range; see Supplementary Table 1*). Shape: Diamond; Font Type: Impact; Font Size: 1; Ellipticity: 0.65; Padding: 1; Rotate: 0.

Supplementary Table 28. Top 10 Subject Categories (WoS) showing frequency range 14-225. Without square root normalisation the skewed data failed to present breath of categories involved.

| **Terms** | **Frequency** |
| --- | --- |
| endocrinology & metabolism | 225 |
| clinical neurology | 178 |
| neurosciences | 141 |
| medicine general & internal | 44 |
| surgery | 23 |
| anesthesiology | 20 |
| biochemistry & molecular biology | 18 |
| medicine research & experimental | 15 |
| multidisciplinary sciences | 14 |
| pharmacology & pharmacy | 14 |

## Web of Science Keyword Plus for Frequent Word

Keyword Plus; 30 Words: Word occurrence by Frequency (*Selected as visual distributed to mitigate large range; see* Supplementary Table 2). Shape: Diamond; Font Type: Impact; Font Size: 0.7; Ellipticity: 0.65; Padding: 1; Rotate: 0.

Supplementary Table 29. Top 10 Keyword Plus showing frequency range 32-99. Square root normalisation was not required as distribution range was constrained.

| **Terms** | **Frequency** |
| --- | --- |
| prevalence | 99 |
| complications | 64 |
| foot ulceration | 51 |
| diagnosis | 48 |
| disease | 44 |
| skin biopsy | 38 |
| population | 35 |
| dysfunction | 34 |
| multicenter | 33 |
| autonomic neuropathy | 32 |

## Co-citation: Sources

Field: Sources | Separator " ; " | Method Parameters: Network Layout: Star for Supplementary Figure 1 and Fruchterman & Reingold for Supplementary Figure 3. Clustering Algorithm: Walktrap. Number of Nodes: 50. Repulsion Force: 0.1. Remove Isolated Nodes: Yes. Minimun Number of Edges: 2. Graphical Parameters: Short Label: Yes. Number of labels: 1000. Label Cex: Yes. Node Shape: Dot. Label Size: 1. Edge Size: 2. Node Shadow: Yes. Edit Nodes: No

## Co-word (Co-occurrence): Keyword Plus and Subject Categories (Web of Science)

Field: Keyword Plus and subject Categories | Method Parameters: Network Layout: Automatic (Fruchterman & Reingold). Clustering Algorithm: Walktrap. Node Coloured by Year: No. Number of Nodes: 50. Repulsion Force: 0.1. Remove Isolated Nodes: Yes. Minimun Number of Edges: 2. Graphical Parameters: Opacity: 0.7. Number of labels: 1000. Label Cex: Yes. Node Shape: Dot. Label Size: 3. Edge Size: 5. Node Shadow: Yes. Edit Nodes: No
