# Supplementary material for: Bibliometric Analysis: Insights Into the Podiatric Medicine Landscape of Diabetic Sensory Peripheral Neuropathy and Genomics
Source: J Foot Ankle Res. 2025 Jul 24;18(3):e70062. doi: 10.1002/jfa2.70062 (PMC12289441; doi:10.1002/jfa2.70062)
Supplement: Supplementary file 8 — Supporting Information S8 [file JFA2-18-e70062-s009.docx]

# Supplementary Files 11 References for supplementary files

1. Garfield E, Sher IH. Key words plus [TM]-algorithmic derivative indexing. *Journal-American Society For Information Science* 1993;44:298-98.

2. Zhang J, Yu Q, Zheng F, et al. Comparing keywords plus of WOS and author keywords: A case study of patient adherence research. *Journal of the Association for Information Science and Technology* 2016;67(4):967-72.

3. Gan Y-n, Li D-d, Robinson N, et al. Practical guidance on bibliometric analysis and mapping knowledge domains methodology – A summary. *European Journal of Integrative Medicine* 2022;56:102203.

4. Yang Z, Algesheimer R, Tessone CJ. A Comparative Analysis of Community Detection Algorithms on Artificial Networks. *Scientific Reports* 2016;6(1):30750.

5. Donthu N, Kumar S, Mukherjee D, et al. How to conduct a bibliometric analysis: An overview and guidelines. *Journal of Business Research* 2021;133:285-96.

6. Arcadia. Declaration on Research Assessment (DORA). *Tools to Advance Research Assessment (TARA)* 2024.

7. Koo M, Lin S-C. An analysis of reporting practices in the top 100 cited health and medicine-related bibliometric studies from 2019 to 2021 based on a proposed guidelines. *Heliyon* 2023;9(6):e16780.

8. Raputova J, Srotova I, Vlckova E, et al. Sensory phenotype and risk factors for painful diabetic neuropathy: a cross-sectional observational study. *PAIN* 2017;158(12):2340-53.

9. Reeves ND, Orlando G, Brown SJ. Sensory-Motor Mechanisms Increasing Falls Risk in Diabetic Peripheral Neuropathy. *Medicina (Kaunas)* 2021;57(5).

10. Santos TRM, Melo JV, Leite NC, et al. Usefulness of the vibration perception thresholds measurement as a diagnostic method for diabetic peripheral neuropathy: Results from the Rio de Janeiro type 2 diabetes cohort study. *Journal of Diabetes and its Complications* 2018;32(8):770-76.

11. Deng P, Shi H, Pan X, et al. Worldwide Research Trends on Diabetic Foot Ulcers (2004–2020): Suggestions for Researchers. *Journal of Diabetes Research* 2022;2022:7991031.

12. Dyck PJ, Kratz KM, Karnes JL, et al. THE PREVALENCE BY STAGED SEVERITY OF VARIOUS TYPES OF DIABETIC NEUROPATHY, RETINOPATHY, AND NEPHROPATHY IN A POPULATION-BASED COHORT - THE ROCHESTER DIABETIC NEUROPATHY STUDY. *Neurology* 1993;43(4):817-24.

13. Young MJ, Boulton AJM, Macleod AF, et al. A multicentre study of the prevalence of diabetic peripheral neuropathy in the United Kingdom hospital clinic population. *Diabetologia* 1993;36(2):150-54.

14. Young MJ, Breddy JL, Veves A, et al. The Prediction of Diabetic Neuropathic Foot Ulceration Using Vibration Perception Thresholds: A prospective study. *Diabetes Care* 1994;17(6):557-60.

15. Feldman EL, Stevens MJ, Thomas PK, et al. A Practical Two-Step Quantitative Clinical and Electrophysiological Assessment for the Diagnosis and Staging of Diabetic Neuropathy. *Diabetes Care* 1994;17(11):1281-89.

16. Tesfaye S, Stevens LK, Stephenson JM, et al. Prevalence of diabetic peripheral neuropathy and its relation to glycaemic control and potential risk factors: The EURODIAB IDDM Complications Study. *Diabetologia* 1996;39(11):1377-84.

17. Adler AI, Boyko EJ, Ahroni JH, et al. Risk factors for diabetic peripheral sensory neuropathy: results of the Seattle Prospective Diabetic Foot Study. *Diabetes Care* 1997;20(7):1162-67.

18. McArthur JC, Stocks EA, Hauer P, et al. Epidermal nerve fiber density: normative reference range and diagnostic efficiency. *Archives of Neurology* 1998;55(12):1513-20.

19. Perkins BA, Olaleye D, Zinman B, et al. Simple Screening Tests for Peripheral Neuropathy in the Diabetes Clinic. *Diabetes Care* 2001;24(2):250-56.

20. Lacomis D. Small-fiber neuropathy. *Muscle & Nerve* 2002;26(2):173-88.

21. Meijer JWG, Smit AJ, Sonderen EV, et al. Symptom scoring systems to diagnose distal polyneuropathy in diabetes: the Diabetic Neuropathy Symptom score. *Diabetic Medicine* 2002;19(11):962-65.

22. Shy ME, Frohman EM, So Y, et al. Quantitative sensory testing: report of the Therapeutics and Technology Assessment Subcommittee of the American Academy of Neurology. *Neurology* 2003;60(6):898-904.

23. Sumner CJ, Sheth S, Griffin JW, et al. The spectrum of neuropathy in diabetes and impaired glucose tolerance. *Neurology* 2003;60(1):108-11.

24. Shun CT, Chang YC, Wu HP, et al. Skin denervation in type 2 diabetes: correlations with diabetic duration and functional impairments. *Brain* 2004;127(7):1593-605.

25. Boulton AJ, Malik RA, Arezzo JC, et al. Diabetic somatic neuropathies. *Diabetes Care* 2004;27(6):1458-86.

26. Gregg EW, Sorlie P, Paulose-Ram R, et al. Prevalence of Lower-Extremity Disease in the U.S. Adult Population ≥40 Years of Age With and Without Diabetes: 1999–2000 National Health and Nutrition Examination Survey. *Diabetes Care* 2004;27(7):1591-97.

27. Boulton AJ, Vinik AI, Arezzo JC, et al. Diabetic neuropathies: a statement by the American Diabetes Association. *Diabetes Care* 2005;28(4):956-62.

28. Devigili G, Tugnoli V, Penza P, et al. The diagnostic criteria for small fibre neuropathy: from symptoms to neuropathology. *Brain* 2008;131(7):1912-25.

29. Lauria G, Hsieh ST, Johansson O, et al. European Federation of Neurological Societies/Peripheral Nerve Society Guideline on the use of skin biopsy in the diagnosis of small fiber neuropathy. Report of a joint task force of the European Fe-deration of Neurological Societies and the Peripheral Nerve Society. *European Journal of Neurology* 2010;17(7):903-e49.

30. Tesfaye S, Boulton AJM, Dyck PJ, et al. Diabetic Neuropathies: Update on Definitions, Diagnostic Criteria, Estimation of Severity, and Treatments. *Diabetes Care* 2010;33(10):2285-93.

31. Lauria G, Bakkers M, Schmitz C, et al. Intraepidermal nerve fiber density at the distal leg: a worldwide normative reference study. *Journal of the Peripheral Nervous System* 2010;15(3):202-07.

32. Backonja MM, Attal N, Baron R, et al. Value of quantitative sensory testing in neurological and pain disorders: NeuPSIG consensus. *PAIN* 2013;154(9):1807-19.

33. Azmi S, Ferdousi M, Petropoulos IN, et al. Corneal Confocal Microscopy Identifies Small-Fiber Neuropathy in Subjects With Impaired Glucose Tolerance Who Develop Type 2 Diabetes. *Diabetes Care* 2015;38(8):1502-08.

34. Themistocleous AC, Ramirez JD, Shillo PR, et al. The Pain in Neuropathy Study (PiNS): a cross-sectional observational study determining the somatosensory phenotype of painful and painless diabetic neuropathy. *PAIN* 2016;157(5):1132-45.

35. Pop-Busui R, Boulton AJ, Feldman EL, et al. Diabetic Neuropathy: A Position Statement by the American Diabetes Association. *Diabetes Care* 2017;40(1):136-54.

36. Iqbal Z, Azmi S, Yadav R, et al. Diabetic Peripheral Neuropathy: Epidemiology, Diagnosis, and Pharmacotherapy. *Clinical Therapeutics* 2018;40(6):828-49.

37. Sopacua M, Hoeijmakers JGJ, Merkies ISJ, et al. Small-fiber neuropathy: Expanding the clinical pain universe. *Journal of the Peripheral Nervous System* 2019;24(1):19-33.

38. Zaharia OP, Strassburger K, Strom A, et al. Risk of diabetes-associated diseases in subgroups of patients with recent-onset diabetes: a 5-year follow-up study. *The Lancet Diabetes & Endocrinology* 2019;7(9):684-94.

39. Rosenberger DC, Blechschmidt V, Timmerman H, et al. Challenges of neuropathic pain: focus on diabetic neuropathy. *Journal of Neural Transmission* 2020;127(4):589-624.

40. Røikjer J, Jensen MH, Vestergaard P, et al. Twenty years with diabetes and amputations: a retrospective population‐based cohort study. *Diabetic Medicine* 2020;37(12):2098-108.

41. Burgess J, Frank B, Marshall A, et al. Early Detection of Diabetic Peripheral Neuropathy: A Focus on Small Nerve Fibres. *Diagnostics* 2021;11(2):165.

42. Røikjer J, Werkman NC, Ejskjaer N, et al. Incidence, hospitalization and mortality and their changes over time in people with a first ever diabetic foot ulcer. *Diabetic Medicine* 2022;39(4):e14725.

43. McCray BA, Diehl E, Sullivan JM, et al. Neuropathy-causing TRPV4 mutations disrupt TRPV4-RhoA interactions and impair neurite extension. *Nature Communications* 2021;12(1):1444.

44. Ziegler D, Tesfaye S, Spallone V, et al. Screening, diagnosis and management of diabetic sensorimotor polyneuropathy in clinical practice: International expert consensus recommendations. *Diabetes Research and Clinical Practice* 2022;186:109063.

45. Ślęczkowska M, Almomani R, Marchi M, et al. Peripheral Ion Channel Genes Screening in Painful Small Fiber Neuropathy. *International Journal of Molecular Sciences* 2022;23(22):14095.

46. Themistocleous AC, Baskozos G, Blesneac I, et al. Investigating genotype-phenotype relationship of extreme neuropathic pain disorders in a UK national cohort. *Brain Communications* 2023;5(2):21.

47. Roikjer J, Croosu SS, Hansen TM, et al. The co-existence of sensory and autonomic neuropathy in type 1 diabetes with and without pain. *Acta Diabetologica* 2023;60(6):777-85.

48. Røikjer J, Croosu SS, Sejergaard BF, et al. Diagnostic Accuracy of Perception Threshold Tracking in the Detection of Small Fiber Damage in Type 1 Diabetes. *Journal of Diabetes Science and Technology*;0(0):19322968231157431.

49. Røikjer J, Croosu SS, Frøkjær JB, et al. Perception threshold tracking: validating a novel method for assessing function of large and small sensory nerve fibers in diabetic peripheral neuropathy with and without pain. *PAIN* 2023;164(4):886-94.

50. Croosu SS, Røikjer J, Mørch CD, et al. Alterations in Functional Connectivity of Thalamus and Primary Somatosensory Cortex in Painful and Painless Diabetic Peripheral Neuropathy. *Diabetes Care* 2022;46(1):173-82.
