# Supplementary material for: Bibliometric Analysis: Insights Into the Podiatric Medicine Landscape of Diabetic Sensory Peripheral Neuropathy and Genomics
Source: J Foot Ankle Res. 2025 Jul 24;18(3):e70062. doi: 10.1002/jfa2.70062 (PMC12289441; doi:10.1002/jfa2.70062)
Supplement: Supplementary file 9 — Figure S1 [file JFA2-18-e70062-s003.docx]

# Supplementary File 4 Co-Citation Expansion


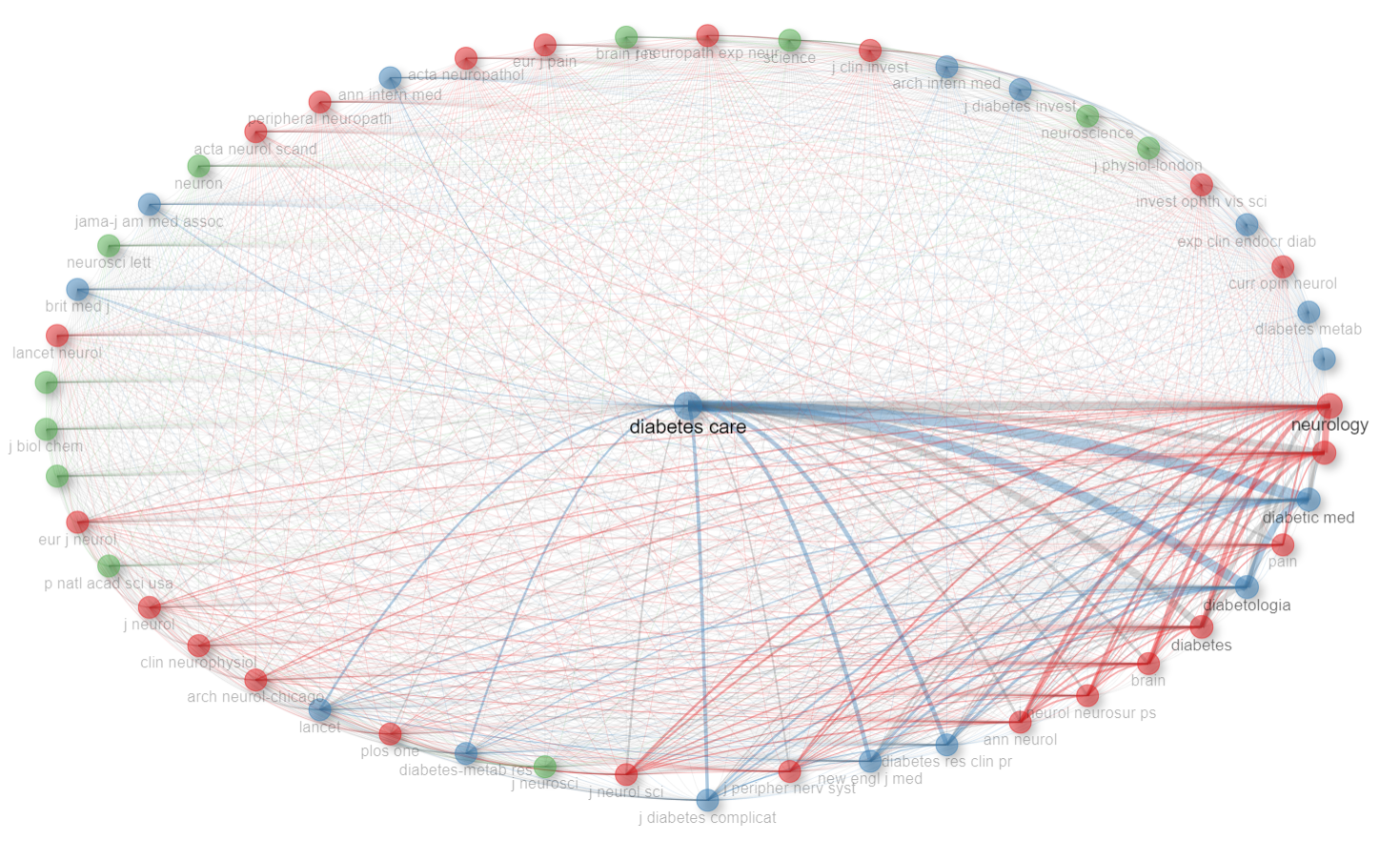


Supplementary Figure 1 Star layout of Co-citation Network Analysis of journals. The three (3) distinct clusters in blue, red, and green are better seen in Supplementary Figure 5 to appreciate in-cluster proximity. Visually the star layout readily provides reader the concentration from core structures within corpus. Diabetes care and Neurology have highest co-citations. Beneath Neurology is Muscle & Nerve, Diabetic Medicine, Pain, Diabetologia, Diabetes, and Brain. Journal of Diabetes and its Complications reside next to Journal of Peripheral Neuropathy. Central tenets are two distinct focuses emerge: diabetes management across the spectrum and clinical neurology and neuroscience, assessment and understanding of pathologies respectively
