# Supplementary material for: Bibliometric Analysis: Insights Into the Podiatric Medicine Landscape of Diabetic Sensory Peripheral Neuropathy and Genomics
Source: J Foot Ankle Res. 2025 Jul 24;18(3):e70062. doi: 10.1002/jfa2.70062 (PMC12289441; doi:10.1002/jfa2.70062)
Supplement: Supplementary file 10 — Figure S2 [file JFA2-18-e70062-s001.docx]

# Supplementary File 5 Dataset Overview


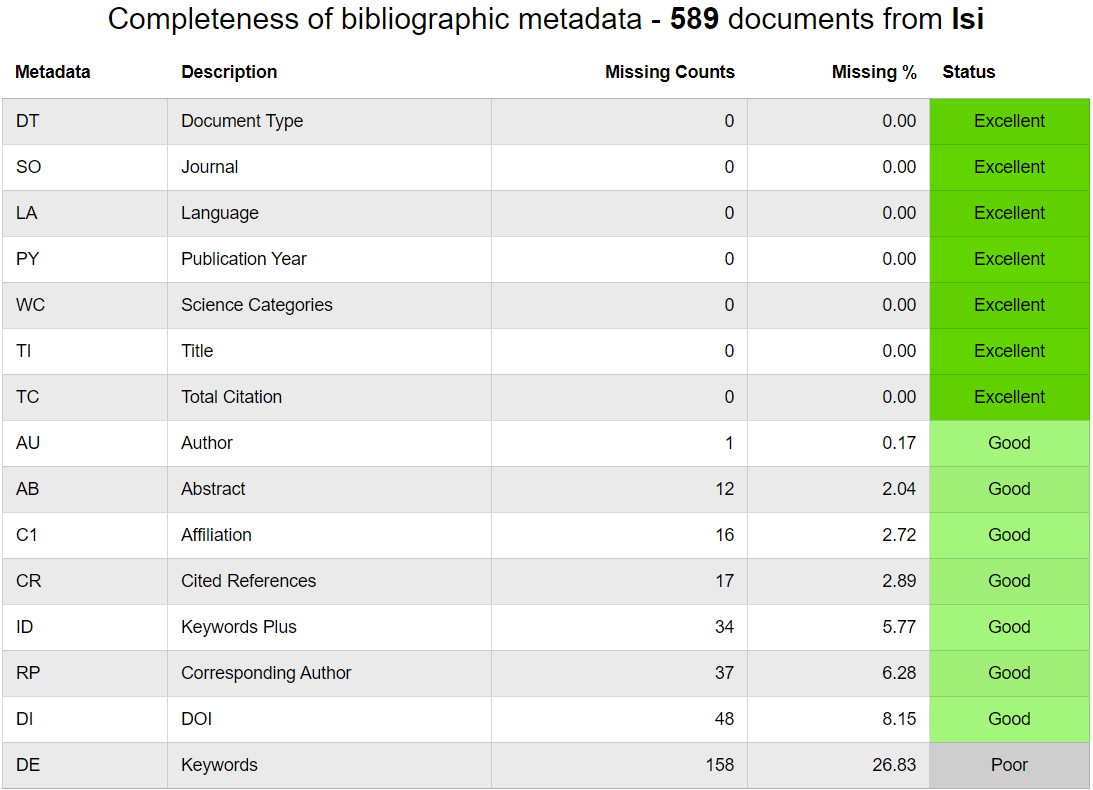


Supplementary Figure 2 Completeness of the n=589 dataset prior to analysis. Only keywords were poor quality with 26.83% missing reducing completeness when exploring themes.

Analysis re-run Monday, 19 February 2024 to reverify elements, settings, and figures.
