# Supplementary material for: Bibliometric Analysis: Insights Into the Podiatric Medicine Landscape of Diabetic Sensory Peripheral Neuropathy and Genomics
Source: J Foot Ankle Res. 2025 Jul 24;18(3):e70062. doi: 10.1002/jfa2.70062 (PMC12289441; doi:10.1002/jfa2.70062)
Supplement: Supplementary file 11 — Table S23 [file JFA2-18-e70062-s006.docx]

# Supplementary File 7 Filters

## Filters: Synonyms and Exclusion

Frequently synonyms of language e.g., fiber and fibre, created a subset that was not representative of the core constituents i.e., anatomical identification across two English speaking countries were not two separate fields. Combined terms consolidated these to represent an informational unit clear without compromising meaning [Supplementary Table 1.

Supplementary Table 23 Inclusion, Exclusion, and Synonyms Terms Table: Excluded from word analysis were terms used in original search strategy and naturally overrepresented.

| **Excluded Terms from Search (Trivial word replication)** |
| --- |
| neuropathy,diabetes,diabetic,peripheral neuropathy,peripheral neuropathies,polyneuropathy,polyneuropathies,sensory,diffuse,sensorimotor,distal,symmetrical,vibration,genomic,genome,gene,genetics,genetic,polymorphism,SNP,mutation,phenotype,phenotyping,phenotypes,assessment,categorisation,categorization,quantitative testing,risk,risk profile,stratification,prognosis,prognostication,prognosticate,personalised medicine,podiatry,podiatrist,podiatric |
| **Additional Excluded terms (evident similarity but missed above terms)** |
| Type,mellitus,risk-factors,corneal confocal microscopy,quantitative sensory testing,diabetic polyneuropathy, diabetic neuropathies,vibration perception threshold, risk factor,risk factors, diabetes mellitus,diabetic peripheral neuropathy,diabetic peripheral neuropathies,diabetic-neuropathy,diabetic-neuropathies,sensory neuropathy,diabetic neuropathy |
| **Combined synonym terms (from current exclusion criterion)** |
| **diabetic peripheral neuropathy**,diabetic peripheral neuropathies,dpn  **diabetic neuropathy,**diabetic neuropathies,diabetic-neuropathies,diabetic-neuropathy  **vibration perception threshold**,vpt  **quantitative sensory testing**,qst  **type 2 diabetes**,type 2 diabetic,type 2 diabetes mellitus  **type 1 diabetes**,type 1 diabetic,type 1 diabetes mellitus  **diabetes mellitus**,diabetes,diabetic,dm  **peripheral neuropathy**,pn,peripheral neuropathies,peripheral-neuropathy,peripheral-neuropathies  **polyneuropathy**,polyneuropathies,  **genomic**,genome  **genetics**,genetic  **polymorphism**,SNP  **phenotype**,phenotyping,phenotypes  **risk**,risk profile,risk factors,risk factor,risk-factors,risk-factor  **prognosis**,prognostication,prognosticate  **podiatry**,podiatrist,podiatric  ***Bold*** Terms are the designated synonym |
| **Combined synonym terms (derived from results indicating overlap)** |
| **Painful neuropathy**,painful neuropathies,painful-neuropathy,painful-neuropathies,neuropathic pain  **Small fibre neuropathy**,small fibre neuroapthies,small-fibre neuropathy,small-fibre neuroapthies,small fiber neuropathy,small fiber neuroapthies,small-fiber neuropathy,small-fiber neuroapthies  ***Bold*** Terms are the designated synonym |
